# Modeling and predicting individual variation in COVID-19 vaccine-elicited antibody response in the general population

**DOI:** 10.1371/journal.pdig.0000497

**Published:** 2024-05-03

**Authors:** Naotoshi Nakamura, Yurie Kobashi, Kwang Su Kim, Hyeongki Park, Yuta Tani, Yuzo Shimazu, Tianchen Zhao, Yoshitaka Nishikawa, Fumiya Omata, Moe Kawashima, Makoto Yoshida, Toshiki Abe, Yoshika Saito, Yuki Senoo, Saori Nonaka, Morihito Takita, Chika Yamamoto, Takeshi Kawamura, Akira Sugiyama, Aya Nakayama, Yudai Kaneko, Yong Dam Jeong, Daiki Tatematsu, Marwa Akao, Yoshitaka Sato, Shoya Iwanami, Yasuhisa Fujita, Masatoshi Wakui, Kazuyuki Aihara, Tatsuhiko Kodama, Kenji Shibuya, Shingo Iwami, Masaharu Tsubokura

**Affiliations:** 1 interdisciplinary Biology Laboratory (iBLab), Division of Natural Science, Graduate School of Science, Nagoya University, Nagoya, Japan; 2 Department of Radiation Health Management, Fukushima Medical University School of Medicine, Fukushima, Japan; 3 Department of General Internal Medicine, Hirata Central Hospital, Fukushima, Japan; 4 Department of Science System Simulation, Pukyong National University, Busan, South Korea; 5 Department of Mathematics, Pusan National University, Busan, South Korea; 6 Medical Governance Research Institute, Tokyo, Japan; 7 Proteomics Laboratory, Isotope Science Center, The University of Tokyo, Tokyo, Japan; 8 Laboratory for Systems Biology and Medicine, Research Center for Advanced Science and Technology, The University of Tokyo, Tokyo, Japan; 9 Medical & Biological Laboratories Co., Ltd, Tokyo, Japan; 10 Department of Virology, Nagoya University Graduate School of Medicine, Nagoya, Japan; 11 Department of Laboratory Medicine, Keio University School of Medicine, Tokyo, Japan; 12 International Research Center for Neurointelligence, The University of Tokyo Institutes for Advanced Study, The University of Tokyo, Tokyo, Japan; 13 Soma Medical Center of Vaccination for COVID-19, Fukushima, Japan; 14 Tokyo Foundation for Policy Research, Tokyo, Japan; 15 Institute of Mathematics for Industry, Kyushu University, Fukuoka, Japan; 16 Institute for the Advanced Study of Human Biology (ASHBi), Kyoto University, Kyoto, Japan; 17 Interdisciplinary Theoretical and Mathematical Sciences Program (iTHEMS), RIKEN, Saitama, Japan; 18 NEXT-Ganken Program, Japanese Foundation for Cancer Research (JFCR), Tokyo, Japan; 19 Science Groove Inc., Fukuoka, Japan; 20 Minamisoma Municipal General Hospital, Fukushima, Japan; Yonsei University College of Medicine, REPUBLIC OF KOREA

## Abstract

As we learned during the COVID-19 pandemic, vaccines are one of the most important tools in infectious disease control. To date, an unprecedentedly large volume of high-quality data on COVID-19 vaccinations have been accumulated. For preparedness in future pandemics beyond COVID-19, these valuable datasets should be analyzed to best shape an effective vaccination strategy. We are collecting longitudinal data from a community-based cohort in Fukushima, Japan, that consists of 2,407 individuals who underwent serum sampling two or three times after a two-dose vaccination with either BNT162b2 or mRNA-1273. Using the individually reconstructed time courses of the vaccine-elicited antibody response based on mathematical modeling, we first identified basic demographic and health information that contributed to the main features of the antibody dynamics, i.e., the peak, the duration, and the area under the curve. We showed that these three features of antibody dynamics were partially explained by underlying medical conditions, adverse reactions to vaccinations, and medications, consistent with the findings of previous studies. We then applied to these factors a recently proposed computational method to optimally fit an “antibody score”, which resulted in an integer-based score that can be used as a basis for identifying individuals with higher or lower antibody titers from basic demographic and health information. The score can be easily calculated by individuals themselves or by medical practitioners. Although the sensitivity of this score is currently not very high, in the future, as more data become available, it has the potential to identify vulnerable populations and encourage them to get booster vaccinations. Our mathematical model can be extended to any kind of vaccination and therefore can form a basis for policy decisions regarding the distribution of booster vaccines to strengthen immunity in future pandemics.

## Author summary

This study investigates the dynamics of antibody responses following COVID-19 vaccination, with the aim of elucidating individual-level variability in immune responses. Using a mathematical model and longitudinal antibody measurements from a vaccination cohort in Fukushima, Japan, we reconstructed the time course of antibody dynamics after vaccination. The study showed that not everyone’s immune system responds equally to the vaccine. Some people may have lower antibody levels after vaccination, which could make them more susceptible to disease. We identified key factors that influence antibody responses, such as age, adverse reactions, comorbidities and medication use, and developed a personalized antibody score to predict individual antibody levels. These factors play a critical role in shaping the immune landscape following vaccination, highlighting the need for tailored approaches to assessing vaccine efficacy and recommending booster doses for vulnerable populations. The development of a personalized antibody score provides a practical tool for healthcare professionals to stratify individuals based on their predicted antibody levels, facilitating targeted interventions to enhance immune protection. The study underscores the importance of considering personal characteristics when assessing vaccine efficacy and suggests potential applications in guiding booster vaccination strategies.

## Introduction

The global distribution of vaccines for coronavirus disease 2019 (COVID-19) and the high vaccine potency and coverage will bring the pandemic caused by severe acute respiratory syndrome coronavirus 2 (SARS-CoV-2) under control. As we learned from the COVID-19 pandemic, vaccination is an important part of a multi-faceted public health response to future pandemic illnesses. An unprecedentedly large volume of high-quality data have been accumulated during the COVID-19 pandemic, and we should use these data to prepare for future pandemics. In particular, given the limited global supply of vaccines during the early phase of a pandemic, determining vaccination priority is important to effectively distribute doses and achieve early disease control. In addition, waning of vaccine efficacy becomes a major concern during vaccination campaigns, as we observed for COVID-19 vaccinations [1,2]. Because a rapid decline in vaccine-elicited antibodies may result in breakthrough infections [3,4], additional, appropriately timed booster vaccinations will be required to maintain a high-level antibody response at both individual and population levels. Thus, to shape an effective vaccination strategy, it is important to quantify the individual-level time course of antibody dynamics and to identify “vulnerable” populations with sustained low antibody titers. Thus, by using a large volume of high-quality data on COVID-19 vaccination, our purpose in this study was to establish an approach to quantifying vaccine-elicited time-course antibody dynamics and predicting individual-level antibody responses with a simple and noninvasive method.

To date, the findings of studies evaluating vulnerable populations for COVID-19 have been biased by the selection of study population. Rather than studying a general population, studies to date have focused on specific subpopulations stratified by variables like age, sex, lifestyle habits, comorbidities, adverse reactions, or medication use [2,3,5–8]. However, variation in vaccine-elicited antibody responses among healthy individuals per se has also been suggested to correlate with breakthrough infection risk [2,9–11]. In fact, although the heterogeneity in antibody responses over time is important for identifying the characteristics of vulnerable populations (in addition to standard risk factors such as age and comorbidities) [2,3,5–8], individual-level variation remains poorly understood. This is also true for viruses beyond SARS-CoV-2, because the dynamics of antibody responses in humans has not been described in detail for any vaccination.

Here we used a mathematical model to describe the process of differentiation from naïve B cells to plasma cells to accurately reconstruct individual vaccine-elicited antibody dynamics. To fit the model, we used longitudinal antibody measurements from non-sequential and sequential blood sampling in the Fukushima vaccination cohort (a community-based cohort in Fukushima, Japan). Of note, because our vaccination cohort consisted of participants from a primarily rural area where the prevalence of COVID-19 was relatively low, the samples from this cohort are ideal for modeling vaccine-elicited antibody dynamics. Thanks to the reconstructed time-course of antibody dynamics, we were able to compare the antibody response at the same time points at an individual level. This overcomes another limitation of current vaccination studies, which can only directly compare antibody titers at a population level rather than at an individual level at specific time points (i.e., date on blood sampling) [2,3,5]. The model parameters describe highly variable individual-level antibody responses, allowing us to partially predict variation in vaccine response on the basis of personal information including age, adverse reactions, comorbidities, and medication use. Furthermore, we devised a personalized antibody score that aims to identify individuals with higher or lower antibody titers from their personal information. Although this score may not be able to detect all individuals, it has the potential to be used by medical practitioners to encourage individuals with low predicted antibody levels to get booster vaccinations. We stress that our approach will be easily applied to reconstruct antibody responses even after the third, fourth and fifth booster doses.

## Results

### The Fukushima vaccination cohort

**Fig 1:**
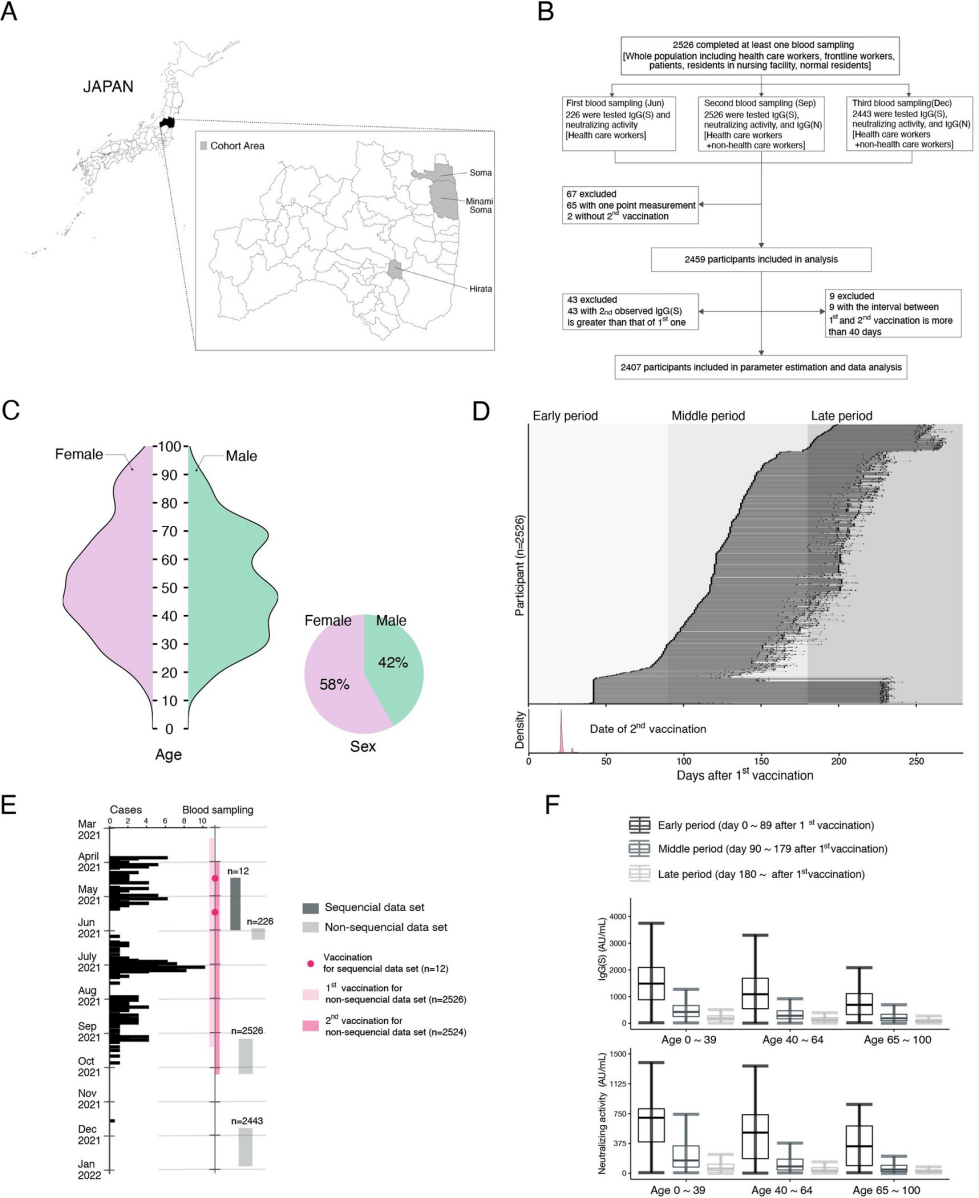
**Characteristics of the Fukushima vaccination cohort: (A)** Locations of Soma city, Minami Soma city, and Hirata village in Fukushima prefecture are described. **(B)** Flowchart of the vaccination cohort along with the number of participants, testing, and inclusion criteria for our analysis are described. **(C)** Age and sex distributions in the cohort are shown. **(D)** Timeline of sample collection for each cohort participant (N = 2,526 participants, 5195 total samples) is described. The timings of blood samplings are indicated in black circles. Shaded areas indicate early (<89 days), middle (90–179 days), and late (>180 days) time periods after the first vaccination. Dates for the second vaccination are shown as the distributions in the bottom panel. **(E)** Vaccination and blood sampling periods in the cohort along with the number of COVID-19 cases (i.e., cases) in Soma city, Minami Soma city, and Hirata village [67,68] are shown. **(F)** Longitudinal IgG(S) and neutralizing activity measured by CLIA are separately plotted by time after the first vaccination and age.

Our vaccination cohort, the Fukushima vaccination cohort, was conducted beginning in April 2021 and consisted of participants from a primarily rural area where COVID-19 prevalence was relatively low: Soma City, Minami Soma City, and Hirata village in Fukushima in Japan (**Fig 1A**). The data used in this study were obtained from April 2021 through December 2021. The participants included health care workers, frontline workers, administrative officers, general residents, and residents of long-term care facilities. In total, 2,526 participants who had been vaccinated with the Pfizer BNT162b2 or Moderna mRNA-1273 vaccine were recruited, and 2,407 participants were included in the final data analysis (see **Fig 1B** and **Methods** for more details). The age and sex distributions of the participants are shown in **Fig 1C**, and the sample characteristics and information on adverse vaccine events stratified by age (with p-values) are provided in **Table 1**. A portion of this cohort was described previously for the time period extending to 6 months after the first dose of mRNA vaccine [12–19].

**Table 1:** Basic demographics for the Fukushima vaccination cohort.

Characteristic	<40 years	40–64 years	≥65 years	Overall	p-value
**Gender**					0.001
Male	338(46.8)	421(38.2)	286(40.8)	1045(41.4)	
Vaccine type*					0.015
BNT162b2 (Pfizer–BioNTech)	651(90.0)	971(88.1)	649(92.6)	2271(89.9)	
mRNA-1273 (Moderna)	0(0.0)	3(0.3)	0(0.0)	3(0.1)	
**Days from 2nd vaccination** (mean [SD])					
1st measurement	106.3[37.7]	104.4[35.1]	103.8[17.2]	104.8[32.0]	0.302
2nd measurement	181.4[37.5]	180.1[35.7]	180.1[16.9]	180.5[32.2]	0.292
**Interval (days) (mean [SD])**	22.6[3.4]	22.8[3.7]	22.2[2.9]	22.6[3.4]	0.002
Blood type*					
A	239(35.7)	427(39.0)	212(35.4)	878(37.2)	<0.001
B	151(22.6)	236(21.6)	140(23.4)	527(22.3)	<0.001
O	213(31.8)	326(29.8)	172(28.7)	711(30.1)	0.367
AB	66(9.9)	105(9.6)	75(12.5)	246(10.4)	0.432
**BMI (kg/m**^**2**^**)** (mean [SD])	23.4[4.6]	23.8[3.8]	23.3[3.5]	23.6[4.0]	0.058
BCG history*	560(83.5)	907(83.1)	412(61.9)	1879(77.4)	0.395
**Smoking**	144(19.9)	253(23.0)	63(9.0)	460(18.2)	<0.001
Drinking Habit**					<0.001
Almost not	403(55.7)	542(49.2)	458(65.3)	1403(55.5)	
Occasionally	247(34.2)	310(28.1)	90(12.8)	647(25.6)	
Everyday	63(8.7)	222(20.2)	127(18.1)	412(16.3)	
Daily Alcohol Consumption**					<0.001
<20g	322(44.5)	382(34.7)	176(25.1)	880(34.8)	
≥20 & <40g	108(14.9)	214(19.4)	87(12.4)	409(16.2)	
≥40 & <60g	20(2.8)	60(5.4)	20(2.9)	100(4.0)	
≥60g	5(0.7)	13(1.2)	2(0.3)	20(0.8)	
**Comorbidities**					
Hypertension	8(1.1)	237(21.5)	432(61.6)	677(26.8)	<0.001
Dyslipidemia	12(1.7)	123(11.2)	146(20.8)	281(11.1)	<0.001
Heart disease	14(1.9)	47(4.3)	140(20.0)	201(8.0)	<0.001
Diabetes	6(0.8)	72(6.5)	110(15.7)	188(7.4)	<0.001
Allergic disease	69(9.5)	95(8.6)	21(3.0)	185(7.3)	<0.001
Asthma	49(6.8)	45(4.1)	28(4.0)	122(4.8)	0.079
Liver disease	11(1.5)	45(4.1)	58(8.3)	114(4.5)	<0.001
Cancer	3(0.4)	35(3.2)	46(6.6)	84(3.3)	<0.001
Gout	5(0.7)	45(4.1)	26(3.7)	76(3.0)	0.001
Thyroid disease	8(1.1)	40(3.6)	11(1.6)	59(2.3)	0.005
Lung disease	12(1.7)	11(1.0)	28(4.0)	51(2.0)	<0.001
Mental disease	17(2.4)	16(1.5)	13(1.9)	46(1.8)	0.739
Rheumatism	2(0.3)	16(1.5)	19(2.7)	37(1.5)	0.006
Kidney disease	6(0.8)	7(0.6)	14(2.0)	27(1.1)	0.089
Anaphylaxis	6(0.8)	7(0.6)	5(0.7)	18(0.7)	0.994
Collagen disease	4(0.6)	6(0.5)	5(0.7)	15(0.6)	0.993
COVID-19 (family)	4(0.6)	5(0.5)	1(0.1)	10(0.4)	0.793
COVID-19	0(0.0)	3(0.3)	4(0.6)	7(0.3)	0.38
Immune deficiency	2(0.3)	4(0.4)	0(0.0)	6(0.2)	0.654
Others	51(7.1)	147(13.3)	189(27.0)	387(15.3)	<0.001
**Drug**					
Steroid	9(1.2)	23(2.1)	26(3.7)	58(2.3)	0.002
NSAIDs	31(4.3)	78(7.1)	82(11.7)	191(7.6)	<0.001
Acetaminophen	8(1.1)	22(2.0)	30(4.3)	60(2.4)	<0.001
Antihistamine	46(6.4)	65(5.9)	43(6.1)	154(6.1)	0.367
Immunosuppressants	6(0.8)	10(0.9)	8(1.1)	24(1.0)	0.432
Biologics	2(0.3)	5(0.5)	4(0.6)	11(0.4)	0.395
Anti-cancer agent	0(0.0)	5(0.5)	5(0.7)	10(0.4)	0.292
**Adverse Reaction**					
Local pain	515(71.2)	684(62.1)	228(32.5)	1427(56.5)	<0.001
Fatigue	511(70.7)	627(56.9)	119(17.0)	1257(49.8)	<0.001
Joint pain	327(45.2)	354(32.1)	90(2.8)	771(30.5)	<0.001
Fever (37.5 degrees or higher)	370(51.2)	308(28.0)	41(5.9)	719(28.5)	<0.001
Headache	321(44.4)	331(30.0)	34(4.9)	686(27.2)	<0.001
Fever (under 37.5 degrees)	137(19.0)	209(19.0)	40(5.7)	386(15.3)	<0.001
Dizziness	57(7.9)	45(4.1)	9(1.3)	111(4.4)	<0.001
Nausea	51(7.1)	41(3.7)	6(0.9)	98(3.9)	<0.001
Diarrhea	30(4.2)	25(2.3)	3(0.4)	58(2.3)	<0.001
Others	40(5.5)	69(6.3)	16(2.3)	125(5.0)	0.003

Values are No. (%) unless noted otherwise. P-values were calculated using Fisher’s exact test. BMI, body mass index; BCG, bacille Calmette-Guérin; NSAIDs, nonsteroidal anti-inflammatory drugs.

*Vaccine type was not included in the multiple regression analysis (**S2 Table**) because only a few participants had received the mRNA-1273 (Moderna) vaccine; blood type and BCG history were not included in the multiple regression analysis because they were not considered relevant (see also section ’Characterizing vaccine-elicited antibody dynamics’).

**Drinking habit and daily alcohol consumption were included in the multiple regression analysis, but are not shown in **Fig 3C** because they have more than two values.

Here we investigated antibody titers in the Fukushima vaccination cohort in individuals sampled longitudinally (serum was collected at 2 or 3 different timepoints) for around 4 to 9 months after the second primary dose of mRNA vaccine (see **Fig 1DE** for details). Notably, the number of SARS-CoV-2 infections in this rural area was extremely low (**Fig 1E**), so that we could minimize the influence of natural breakthrough infections. Compared with some of the largest cohorts in the world [1,5,20–23], the Fukushima vaccination cohort is community-based, includes non-health workers, has very few dropouts among more than 2,000 individuals who were consecutively sampled (only 3.3%), includes all necessary information for all participants, and includes measures of several modalities of antibody titers including neutralizing activity.

We performed chemiluminescent immunoassay (CLIA) to measure antibody titers as a measure of humoral immune status after the first COVID-19 vaccination (i.e., a total of 5195 IgG(S), 5195 neutralization activity, and 4969 IgG(N) assays were performed) (**Methods**). **Fig 1F** shows the overall profile of IgG(S) and neutralization activity against the Wuhan strain in this study. We investigated longitudinal data for IgG(S) in the same individuals because IgG(S) covers a wider range of antibody responses and is more sensitive than neutralization activity. In fact, these two measurements are highly correlated with each other (correlation coefficient of 0.93) (**S1 Fig**), and previous studies showed that neutralizing antibody and IgG(S) titers correlate with vaccine-mediated protection, even against variants of concern (i.e., vaccine efficacy) [9,24,25]. This is because vaccines containing the original Wuhan virus spike protein induce variant-reactive memory B cells targeting multiple variants of concern, including the Omicron variant [26]. To prepare for a rapid response to an early phase of a future pandemic, we hereafter investigated longitudinal data for IgG(S) titers against the ancestral strain in the same individuals, which we used as a biomarker for vaccine-elicited immune response at the beginning of a COVID-19 vaccination program, and we used these data to develop an approach for establishing an "antibody score".

### Deriving measures of peak, duration, and area under the curve of vaccine-elicited antibody dynamics

**Fig 2:**
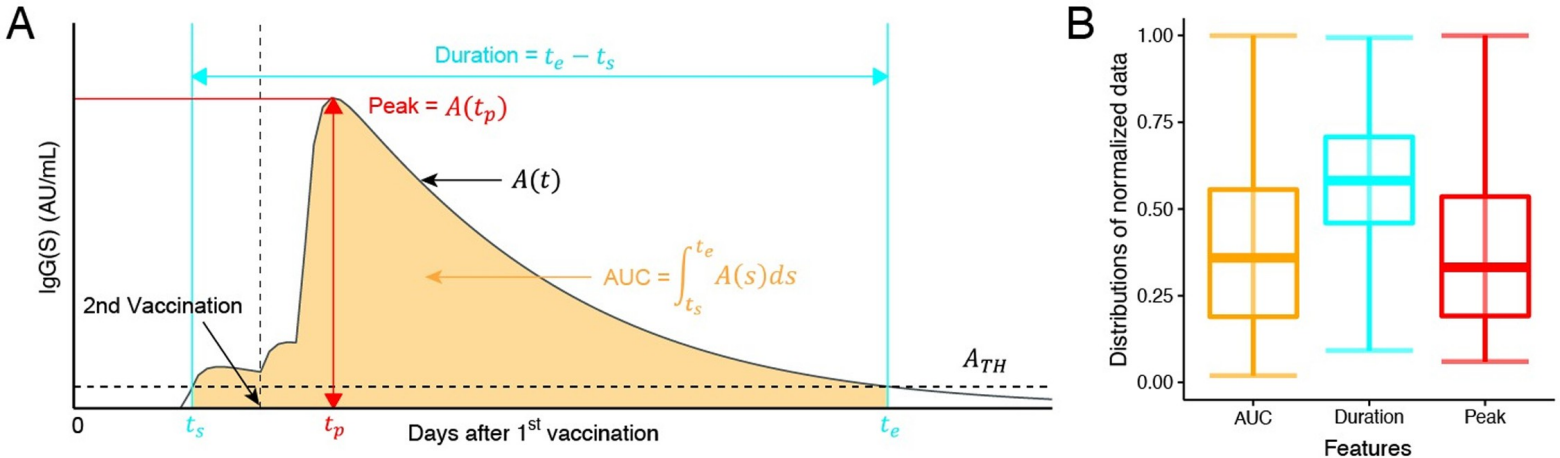
**Quantifying vaccine-elicited antibody dynamics: (A)** Vaccine-elicited antibody response after the first vaccination (i.e., *t* = 0) is described with the following “features”: the peak (*A*(*t*_*p*_)), duration (*t*_*e*_ − *t*_*s*_), and AUC $\left(\int_{t_{s}}^{t_{e}}A(s)\;ds\right)$ of the antibody titers. The vertical and horizontal dashed lines correspond to the date of the second vaccination and the arbitrary threshold (*A*_TH_) for calculating the duration and AUC, respectively. **(B)** Distributions of the extracted features from the reconstructed antibody dynamics (i.e., the peak, duration, and AUC) for 2,407 participants are plotted. The dataset for each distribution was normalized by the value corresponding to the 95th percentile of data values, and data with values larger than this value were removed to improve the visibility of the figure.

We developed a mathematical model describing the vaccine-elicited antibody dynamics to evaluate the impact of primary two-dose COVID-19 vaccination on rapid immunity at the individual level. We fully reconstructed the dynamics of IgG(S) titers after the first vaccination for 2,407 individuals in the Fukushima vaccination cohort in **S2 Fig** (see **Methods** in detail). Of note, we validated our mathematical model and the reconstructed antibody titer curves using independent datasets (see **Methods** for details). Then we extracted the “features” described in **Fig 2A** for each individual: the peak, duration, and area under the curve (AUC) of the reconstructed antibody dynamics. To quantify these features, we here assumed *A*_TH_ = 100, and determined *t*_*s*_ and *t*_*e*_ corresponding to the time for the antibody titer to be greater than and smaller than *A*_TH_, respectively. *t*_*s*_ and *t*_*e*_ are calculated as the minimum and maximum values of the time, respectively, during which the reconstructed IgG(S) remains above *A*_TH_. Therefore, the duration and AUC of the antibody titer are formulated by *t*_*e*_ − *t*_*s*_ and $\int_{t_{s}}^{t_{e}}A(s)\;ds$, respectively. In addition, defining *t*_*p*_ to be the time for the antibody titer to reach its peak, the peak titer is *A*(*t*_*p*_). For 89 individuals whose peak titer was below *A*_TH_, the duration and AUC were both determined to be 0. In **Fig 2B**, we summarized distributions of the AUC, duration, and peak for 2,407 participants. Note that a similar trend was obtained under different *A*_TH_. These features allowed us to quantitatively compare vaccine-elicited antibody dynamics among the participants (see next section).

### Characterizing vaccine-elicited antibody dynamics

**Fig 3:**
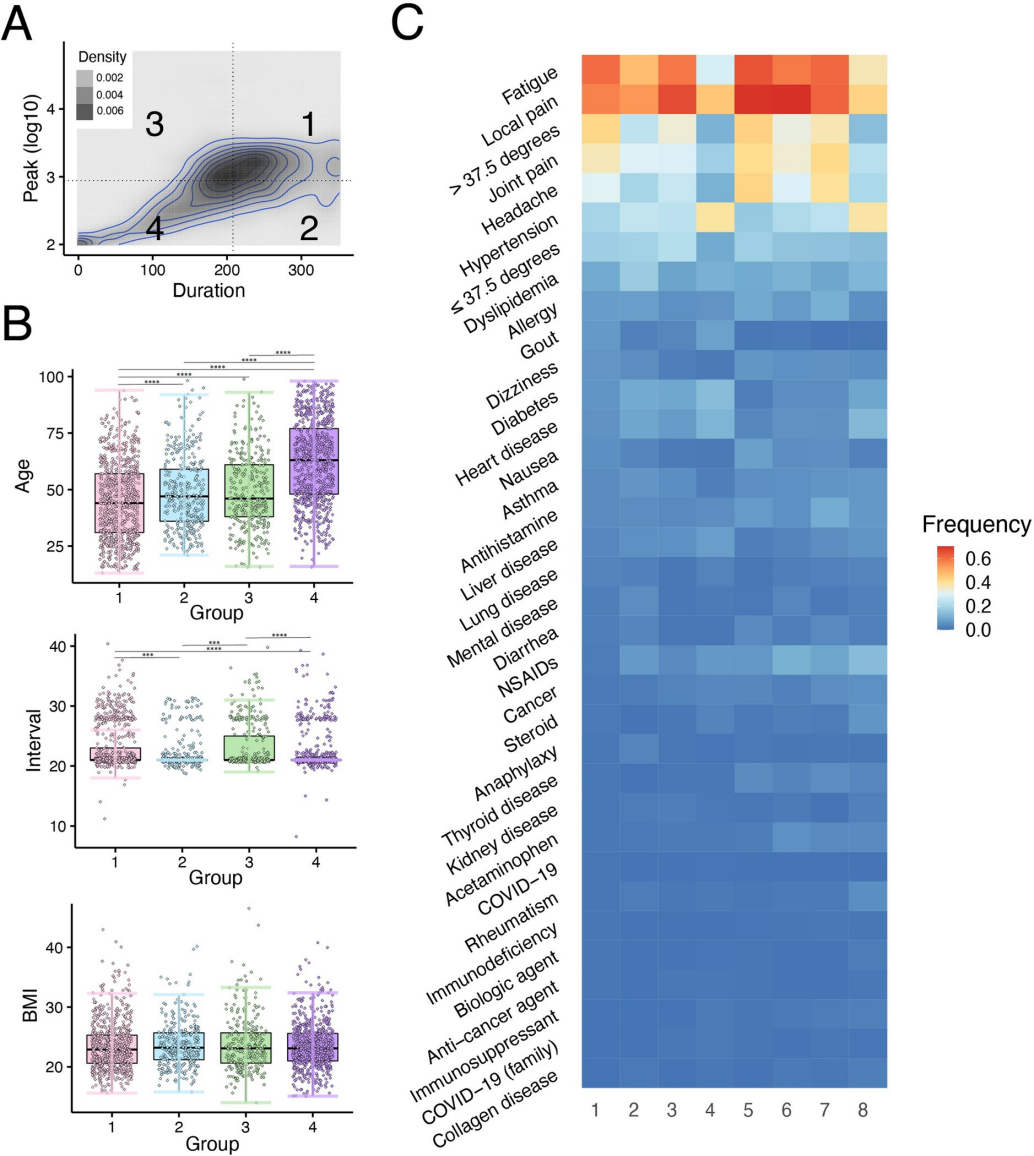
**Characterizing and scoring vaccine-elicited antibody dynamics: (A)** The peak-duration plane was divided into four quadrants (groups 1 to 4). The cutoff values for the peak (log10) and the duration were 2.94 and 208, respectively. The distribution of 2,407 participants on the plane is shown as a density plot. **(B)** Distributions of age, BMI and the interval between the two vaccine doses in groups 1 to 4 are plotted. **(C)** The frequency of each categorical variable in groups 1 to 4, stratified by gender, is shown as a heatmap. In the figure, 1, 2, 3, 4 refer to groups 1–4 in men, whereas 5, 6, 7, 8 refer to groups 1–4 in women.

To see how individual background factors contributed to the three features, we divided the peak-duration plane into four quadrants (groups 1–4) by taking the median values of peak and duration of titer as cutoffs (**Fig 3A**). We collected basic demographic and health information from the participants, including underlying medical conditions, adverse reactions to vaccinations, and medications, as described in **Table 1**. We then investigated whether there were differences in these basic variables among the four groups.

We first analyzed continuous variables such as age, BMI, and the interval between the two vaccinations. We found that group 1 (high peak, long duration) had the youngest participants on average, while group 4 (low peak, short duration) had the oldest participants on average (**Fig 3B** top). Groups 1 and 3 (high peak) had longer intervals between the two vaccinations than groups 2 and 4 (low peak) (**Fig 3B** middle). In contrast, there was no significant difference in BMI among the four groups (**Fig 3B** bottom). We next visualized how categorical variables differed among the four groups, stratified by gender (**Fig 3C**). In the figure, 1, 2, 3, 4 refer to groups 1–4 in men, whereas 5, 6, 7, 8 refer to groups 1–4 in women. Group 4 showed higher frequencies of underlying medical conditions (hypertension, diabetes, rheumatism, heart disease, collagen disease, liver disease) and use of medications (steroids, NSAIDs, immunosuppressants) as well as lower frequencies of adverse reactions to vaccinations (local pain, fever, fatigue, headache, joint pain, nausea).

We further performed multiple regression analysis to see whether each of the three features could be explained by the participants’ demographic and health information (**S2 Table**). The obtained models for the logarithm of the peak and the duration and logarithm of AUC had *R*^2^ values of 0.215, 0.267 and 0.217, respectively. The variables that significantly influenced the peak were age, the interval between vaccinations, dyslipidemia, fever over 37.5 degrees, and gender; for duration, they were age, fever, smoking, steroids, immunosuppressants, and dyslipidemia; and for the AUC, they were age, immunosuppressants, steroids, the interval between vaccinations, dyslipidemia, fever over 37.5 degrees, kidney disease, and rheumatism. Hence, all three features were partially explained by the basic demographic and health information. We note that blood groups (A, B, O, AB) and BCG vaccination history were not predictors of these features [27,28]. In fact, there were no significant differences between BCG vaccinated and unvaccinated individuals in peak (*p* = 0.892), duration (*p* = 0.521) and area under the curve (*p* = 0.873). There were also no significant differences between blood groups in peak (*p* = 0.850), duration (*p* = 0.211) and area under the curve (*p* = 0.200).

### Deriving a personalized antibody score

**Fig 4:**
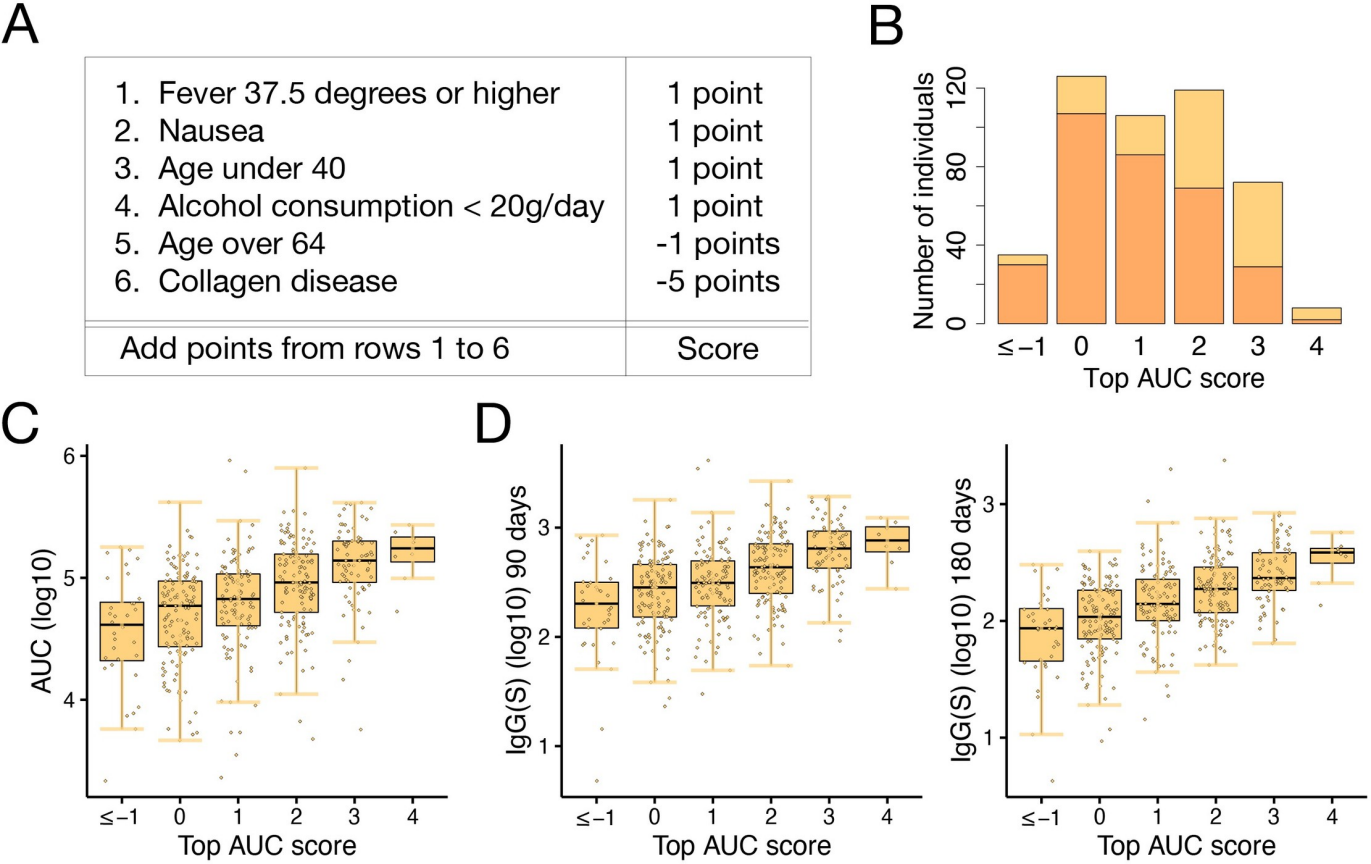
**Scoring vaccine-elicited antibody dynamics: (A)** Metric for calculating the “top AUC score,” i.e., a score to identify individuals with AUC in the top third of the population, is calculated. **(B)** Distribution of the top AUC scores in the test dataset is shown: 35, 126, 106, 119, 72, and 8 individuals had scores of -1 or less, 0, 1, 2, 3, and 4, respectively. Those in the top third of the test dataset are shown in yellow, and those not in the top third are shown in orange. The ratio of individuals with AUC in the top third of the test dataset increased as the top AUC score increased. **(C)** The average AUC tended to increase as the top AUC score increased. **(D)** The top AUC score was correlated with the IgG(S) titers 90 or 180 days from the second vaccination, calculated from the mathematical model.

Recently, a systematic approach to fit optimized scores with mixed-integer nonlinear programming was proposed [29]. Combining the demographic and health information in **Table 1**, we devised a simple score aimed at roughly estimating their antibody status. We chose the AUC as a representative feature of individual antibody status because it reflects both the early and the late phases of antibody dynamics. We here constructed two types of scores to cover the whole range of AUC as follows: a score to predict whether an individual’s AUC is in the top third of the population (i.e., top AUC score, **Fig 4A**) and a score to predict whether an individual’s AUC is in the bottom third (i.e., bottom AUC score, **S3 Fig A**).

We first divided our dataset into training and test datasets. The training dataset consisted of participants in Minami Soma City and Hirata Village (1935 individuals), and the test dataset consisted of participants in Soma City (466 individuals). Six individuals who did not fully answer the questionnaire were excluded from the datasets. We used the training dataset for fitting the scores. The algorithm searches the space of linear combinations of factors with integer coefficients from -5 to +5 to find the best combination to differentiate the population in the top third of the training dataset from the rest (or the bottom third from the rest) (**Fig 4A**). We then assessed the performance of these scores in the test dataset, that is, we tested whether the scores just created could differentiate the population in the top third of the test dataset from the rest (or the bottom third from the rest).

The top AUC scores in the test dataset were between -1 and 4, except for two individuals with scores of -6 and -5, respectively (**Fig 4B** and **S3 Table**). The 8 individuals with scores of 4 (shown in yellow and orange) included 6 individuals (shown in yellow) whose AUC belonged to the top third of the test dataset population (75.0%). The 72 individuals with a score of 3 included 43 individuals with AUC in the top third (59.7%). The 119 individuals with a score of 2 included 50 individuals with AUC in the top third (42.0%). The 106 individuals with a score of 1 included 20 individuals with AUC in the top third (18.9%). The 126 individuals with a score of 0 included 19 individuals with AUC in the top third (15.1%). The 35 individuals with a score of -1 or less included 5 individuals with AUC in the top third (14.3%). Thus, the higher an individual’s top AUC score, the more likely they were to belong to the top third of the population and to have a higher AUC as well (**Fig 4C**). For example, if a healthy 30-year-old male who drinks two bottles of beer every day had a fever of 37.7 degrees but no nausea after vaccination, his score would be calculated as 1+1 = 2, indicating that his AUC is in the top third with a probability of 42.0%. The top AUC score was also well correlated with IgG(S) titers 90 or 180 days from the second vaccination (**Fig 4D**). When the threshold is set to 1.5, the top AUC score has an accuracy of 69.1%, a precision of 49.7%, and a recall of 69.2%, evaluated on the test dataset (see **Methods** for calculation methods).

On the other hand, we calculated the bottom AUC scores in the test dataset, which were between -4 and 2, except for one individual with a score of 3 (**S3 Fig B** and **S3 Table**). We confirmed similar trends. For example, the 58 individuals with a score of 1 included 40 individuals with AUC in the bottom third (69.0%). Thus, the higher an individual’s bottom AUC score, the more likely they were to belong to the bottom third of the population and have a lower AUC as well (**S3 Fig C**). The bottom AUC score was also inversely correlated with IgG(S) titers 90 or 180 days from the second vaccination (**S3 Fig D**). When the threshold is set to -1.5, the bottom AUC score has an accuracy of 61.2%, a precision of 50.7%, and a recall of 80.5%, evaluated on the test dataset. Note that these AUC scores are useful for detecting the top and bottom populations rather than the middle population, given the relatively low frequency of some of the items listed (collagen disease, immunosuppressant, rheumatism). In fact, when we created a "middle AUC score" to detect individuals with AUC in the middle third (**S10 Fig**), 92.7% of individuals in the test dataset had the same score of 0 (**S3 Table**), suggesting that this middle AUC score is not very useful. Note also that even the top and bottom AUC scores will not be able to detect all individuals with top or bottom titer levels, and there will be false positives and false negatives as shown in **S3 Table**.

We also created a scoring for groups 1 (with high peak and long duration) and 4 (with low peak and short duration) in **Fig 3A**. Group 1 score had similar items to the top AUC score, and group 4 score had similar items to the bottom AUC score (**S11 Fig**). These results show that the personalized AUC score can provide a reasonable estimate of an individual’s antibody status, especially for the top and bottom populations, allowing the individual to make informed decisions about disease prevention.

## Discussion

In this study, we created a personalized antibody score that can be used as a basis for identifying populations with low sustained antibody titers. To derive an optimal score, we used a mathematical model of antibody production in response to two-dose mRNA vaccinations and reconstructed the vaccine-elicited antibody dynamics of 2,407 participants from the Fukushima vaccination cohort. In particular, we highlighted that the reconstructed best-fit antibody titer curves perfectly predicted the additional timepoint data sampled from 120 of the 2,407 participants. Our mechanism-based mathematical modeling, in contrast to the statistical modeling used in recent reports [30,31], enables a biologically accurate description and precise comparison of antibody dynamics. The parameters of the estimated dynamics showed a large variation spanning two orders of magnitude. This variation was partially explained by individual characteristics like age, sex, the interval between the two vaccine doses, adverse reactions, comorbidities, and medications taken. This result is consistent with previous studies reporting age, sex, vaccine interval, and comorbidities as factors affecting antibody titers [5,32].

Our antibody scores can be easily calculated from individual demographic and health information, yet identify participants with high and low antibody titers (AUC of the IgG(S) titers) to a certain extent. Given the pleiotropic aspect of humoral immunity, it is surprising that a score consisting of several questionnaire items can provide predictions. The score showed that COVID-19 infection history and adverse reactions positively affect the AUC, whereas age and immunologically compromised conditions negatively affect the AUC. These positive and negative factors are consistent with previous studies [5,19,33–35]. In addition, autoimmune diseases have been reported to be risk factors for breakthrough infections [36,37]. Considering that individual antibody titers are partially predictive of the likelihood of breakthrough infections [11,38], this suggests that our antibody score has potential to be used as a risk score for breakthrough infections. On the other hand, the antibody score also has some similarities with COVID-19 severity scores [39–43]. In fact, both include age as a factor likely to lead to low antibody titers and critical illness, both of which may be related to a defective immune system, as observable in cytokine signatures [44,45] or immunoglobulin responses [46,47]. However, whereas COVID-19 severity scores use the results of laboratory tests and clinical symptoms to assess the patient’s condition in the hospital, our antibody score can be easily calculated on the basis of questionnaire responses provided by individuals (i.e., not limited to patients) themselves.

So far, 13.01 billion doses of COVID-19 vaccines have been administered globally, and 2.51 million are now administered each day [48]. Whereas 68.5% of the world population has received at least one dose of a COVID-19 vaccine, 24.6% of people in low-income countries have received at least one dose as the result of disparities in global access to vaccines [49,50]. For example, only a small percentage of persons have received one vaccine dose in African countries [51]. Although the third and fourth doses of COVID-19 vaccines have already begun to be offered, vaccine uptake after the primary two doses is challenged by (booster) vaccine hesitancy, waning of vaccine coverage due to painful adverse reactions [49], and a drop-off in interest in COVID-19 [52]. Even during the unprecedented but successful worldwide vaccination programs for COVID-19, we experienced difficulty in the global distribution of vaccine doses. One of the lessons we learned was the importance of identifying vulnerable populations to achieve early control of infectious disease. In future pandemics, the score that we have developed may play an important role in identifying vulnerable populations.

There are limitations to this study that we can improve for a better understanding of the role of vaccination. First, we measured IgG(S) and neutralization activity against the Wuhan strain as the humoral immune status, and analyzed IgG(S) because of the high correlation between these measurements. Although it is reported that antibodies induced by infection with the ancestral SARS-CoV-2 strain and/or the first of the primary two-dose vaccinations show cross-neutralization of variants from Alpha to Omicron BA.1 [53,54], further analysis of antibody responses (in particular, for neutralization) against variants of concern may be important to extend our scoring to prediction of vaccine efficacy for corresponding strains. Although our current score is limited in its detection capacity, the creation of such a personalized antibody score in the future by using individual-level demographic and health information will be an important tool for designing optimal vaccination strategies in future pandemics (not only COVID-19 but also other infectious diseases). Second, although one of the advantages of the Fukushima vaccination cohort is the extremely low number of natural infections, and that we can evaluate antibody responses minimizing the effect of infection, large portions of the population have been infected with COVID-19 in many parts of the world. In addition, the third and fourth doses of COVID-19 vaccines have already begun to be administered. Our primary purpose in this study was to establish an integrated framework for scoring, but an extended score considering breakthrough infection and booster vaccinations is worth evaluating. Another limitation is that the model fitting was based on antibody measurements and the antibody score was solely based on information available from the questionnaire. This is reflected in the rather low *R*^2^ values (around 0.2–0.3) of the regression models of the antibody features. Therefore, further refinement of the score using additional information will be worthwhile. It is worth mentioning that the immune system is affected by multiple factors, including genetics, the environment (such as cohabitation), and markers of metabolic health [55–58], all of which likely influence individual antibody status but were not considered here. The biological determinants of antibody variation will be further revealed in future studies addressing not only B cell subsets but the whole immune system encompassing adaptive as well as innate immunity.

In conclusion, quantifying the variability in antibody dynamics can be a basis for policy decisions regarding the distribution of booster vaccines to strengthen immunity [59] or the use of oral antiviral drugs for the treatment of breakthrough infections [60]. As we learned from the COVID-19 pandemic, determining the priority for (booster) vaccines is an important public health concern given a limited global supply of vaccines at the beginning of a pandemic. For example, since we have data on what percentage of people with certain top/bottom AUC scores have low antibody levels, we can estimate how many people should be prioritized for booster vaccination based on these scores. Thus, the score developed here can be used to estimate and allocate necessary vaccination resources to “priority groups” for vaccination [61,62] in addition to the standard prioritized population [1,2,5,6]. Moreover, the score may be used by medical practitioners to encourage individuals with low predicted antibody levels to get (booster) vaccinations. However, we caution that our current score does not identify all individuals who need booster vaccinations and should not be the sole resource for guiding individual decisions. Taking advantage of our Fukushima vaccination cohort, we will further evaluate the impact of booster vaccinations and post-vaccination infections on personalized antibody scores. It is important that we make use of the lessons learned from COVID-19 vaccination to combat future infectious diseases.

## Methods

### Ethics statement

The study was approved by the ethics committees of Hirata Central Hospital (number 2021-0611-1) and Fukushima Medical University School of Medicine (number 2021–116). Written informed consent was obtained from all participants individually before the survey.

### Participant recruitment and sample collection

The candidates were mainly recruited from Hirata village, Soma city, and Minamisoma city in rural Fukushima prefecture. We conducted non-sequential blood sampling and sequential blood sampling. A total of 2526 individuals participated in non-sequential blood sampling (**Fig 1E**). Health care workers, frontline workers, and administrative officers from each municipality were intentionally recruited to keep the cohort size large and the dropout rate low. Although most of the health care workers, frontline workers, and administrative officers were under the age of 65, relatively healthy community-dwelling older adults living in the community and in long-term care facilities were also recruited to maintain a wide age range for the cohort. Blood sampling was performed once during each period in June, September, and November 2021, respectively. The first vaccine dose was administered between March 10 and August 20, 2021, and the second dose between March 31 and September 14. The median (interquartile range) interval for the two-dose vaccination was 21 days. A total of 226 health care workers participated in the first blood sampling between May 31 and June 6, 2021. A total of 2526 individuals participated in the second blood sampling between September 8 and October 8, 2021. A total of 2443 individuals participated in the third blood sampling between November 21 and December 25, 2021. In contrast, 12 health care workers participated in the sequential blood sampling, and their vaccination and blood sampling schedule are shown in **Fig 1E**. Note that the 12 health care workers (described in **S5 Fig A**) with sequential blood sampling were not included in the non-sequential population of 2526 participants. In conclusion, of the total 2526 participants, those eligible for analysis were 2459 participants who completed the second vaccination and at least two blood samplings.

Out of these 2459 participants, 43 individuals had higher IgG(S) titers on the second measurement than on the first, although their IgG(N) titers were negative (meaning no infection history). We speculated that this was due to measurement error and did not attempt model fitting. Furthermore, 9 individuals who had an interval of longer than 40 days between the first and the second vaccination were also removed. Because most of our participants had an interval of 21 or 28 days and there were very few cases of longer intervals, we decided that there were not enough data to reliably perform predictions among the participants with longer intervals. This was a conservative decision to ensure the high quality of our model fitting. As a result, we performed parameter estimation on the remaining 2407 participants (**Fig 1B**).

Information on sex, age, daily medication, medical history, date of vaccination, adverse reaction after vaccination, type of vaccination, blood type, bacillus Calmette–Guérin (BCG) vaccine history, smoking habits, and drinking habits was retrieved from the paper-based questionnaire (summarized in **Table 1**). All blood sampling was performed at the medical facilities with 8 mL, and serum samples were sent to The University of Tokyo.

### SARS-CoV-2-specific antibody measurement

All serological assays were conducted at The University of Tokyo. Specific IgG (i.e., IgG(S)) and neutralizing activity against the Wuhan strain were measured as the humoral immune status after COVID-19 vaccination. Specific IgG antibody titers (IgG(N)) were used to determine past COVID-19 infection status. Chemiluminescent immunoassay with iFlash 3000 (YHLO Biotech, Shenzhen, China) and iFlash-2019-nCoV series (YHLO Biotech, Shenzhen, China) reagents were used in the present study. The measurement range was 2–3500 AU/mL for IgG(S) and 4–800 AU/mL for neutralizing activity. For neutralizing activity, AU/mL×2.4 was used to convert to International Units (IU/mL); for IgG(S), AU/mL×1.0 was used to convert to binding antibody units (BAU/mL). The testing process was as per the official guideline. Quality checks were conducted every day before starting the measurement.

### Modeling vaccine-elicited antibody dynamics

We developed a mathematical model describing COVID-19 vaccine-elicited antibody dynamics to evaluate the impact of primary two-dose COVID-19 vaccination on rapid immunity at the individual level and reconstructed the best-fit antibody titer curves of 2,407 participants in the Fukushima vaccination cohort. Here we explain the derivation and formulation of the mathematical model in detail.

#### (i) Vaccination-elicited antibody dynamics after the first dose

After the first vaccination, naïve B cells encounter antigens and differentiate into short-lived antibody-secreting cells (ASCs), plasmablasts, germinal center (GC) B cells, or GC-independent memory B cells depending on BCR affinity for their cognate antigen [63]. Then, the GC B cells undergo rapid proliferation with somatic immunoglobulin hypermutation and subsequently differentiate into GC-dependent memory B cells or long-lived antibody-secreting cells, which are plasma cells with immunoglobulin class switching. To describe this antigen-specific B cell expansion and the induction of antibody-secreting cells and memory B cells after the first vaccination (**S4 Fig A**), we developed a simple but quantitative mathematical model as follows:

$$\frac{dM_{1}(t)}{dt}=\left\{\begin{array}{cc} 0 & \left(t<\tau_{1}\right)\\ -dM_{1}(t) & {\left(t\geq \tau_{1}\right)}^{\prime } \end{array}\right.$$ (1)

$$\frac{dB(t)}{dt}=P_{1}(t)\frac{M{\left(t\right)}^{m}}{K^{m}+M{\left(t\right)}^{m}}-\mu B(t),$$ (2)

$$\frac{dA(t)}{dt}=pB(t)-cA(t),$$ (3)

where the variables *M*(*t*) = *M*_1_(*t*), *B*(*t*), and *A*(*t*) are the amount of mRNA inoculated by the vaccination, the number of antibody-secreting cells, and the antibody titers at time *t*, respectively. The parameters *τ*_1_ and *d* represent the timing of the vaccination and the decay rate of mRNA. We here considered *D*_1_ to be the inoculated dose of mRNA by the vaccination, that is, *M*_1_(*τ*_1_) = *D*_1_.

Because the data we used here were limited (i.e., only time-course vaccine-elicited IgG(S) titers), one compartment of B cells including heterogeneous cell populations that produce antibodies (i.e., short-lived and long-lived antibody-secreting cells) was assumed. Therefore, we modelled the average B cell population dynamics in Eq (2), where the product of *P*_1_(*t*) and *M*(*t*)^*m*^/(*M*(*t*)^*m*^ + *K*^*m*^) represents the average *de novo* induction of the antibody-secreting cells. *P*_1_(*t*) is a step function defined as *P*_1_(*t*) = *P*_1_ for *τ*_1_ + *η*_1_ ≤ *t*, where *η*_1_ is the delay of induction of antibody-secreting cells after vaccination: otherwise *P*_1_(*t*) = 0. The parameters *m*, *K*, and *μ* correspond to the steepness at which the induction increases with increasing amount of mRNA (i.e., the Hill coefficient), the amount of mRNA satisfying *P*_1_/2, and the average decay rate of the antibody-secreting cell compartment, respectively. The other parameters, *p* and *c*, represent the antibody production rate and the clearance rate of antibodies, respectively.

#### (ii) Vaccination-elicited antibody dynamics after the second dose

After the second vaccination, memory B cells are reactivated by re-exposure to the antigen. Some differentiate into short-lived antibody-secreting cells (plasmablasts) or memory B cells outside the GC. Others enter the GC to become secondary GC B cells. Subsequently, these secondary GC B cells differentiate into GC-dependent memory B cells or long-lived antibody-secreting cells (plasma cells). To describe these recall B cell responses and their secretion of antibody after the second vaccination (**S4 Fig B**), we modified the above mathematical model, Eqs (1–3), as follows:

$$\frac{dM_{2}(t)}{dt}=\left\{\begin{array}{cc} 0 & \left(t<\tau_{2}\right)\\ -dM_{2}(t) & \left(t\geq \tau_{2}\right) \end{array}\right.,$$ (4)

$$\frac{dB(t)}{dt}=P_{2}(t)\frac{M{\left(t\right)}^{m}}{K^{m}+M{\left(t\right)}^{m}}-\mu B(t),$$ (5)

$$\frac{dA(t)}{dt}=pB(t)-cA(t),$$ (6)

where *M*_2_(*t*) is the amount of mRNA by the vaccination inoculated at *τ*_2_ satisfying *M*_2_(*τ*_2_) = *D*_2_. In addition, *P*_2_(*t*) = *P*_1_ and *M*(*t*) = *M*_1_(*t*) for *τ*_2_≤*t*<*τ*_2+_*η*_2_, *P*_2_(*t*) = *P*_2_ and *M*(*t*) = *M*_1_(*t*) + *M*_2_(*t*) for *τ*_2_ + *η*_2_ ≤ *t*, where *η*_2_ is the delay of induction of antibody-secreting cells after vaccination; otherwise *P*_2_(*t*) = 0. Here we ignored the GC-independent memory B cells induced by the first vaccination because of their minor effect. In general, once reactivated, memory B cells can re-enter the GC more rapidly than naïve B cells, and therefore the secondary antibody responses are much faster and larger (i.e., *η*_1_ >*η*_2_ and *P*_1_ < *P*_2_, respectively). In the main recall immunity, the quantity and quality of memory B cells established by the first vaccination is included in *P*_2_.

#### (iii) Mathematical model for data fitting

Since the clearance rate of antibody is much larger than the decay of antibody-secreting cells (i.e., *c* ≫ *μ*), we made a quasi-steady state assumption, *dA*(*t*)/*dt* = 0 dV(t)/dt = 0, and replaced Eqs (3) and (6) with *A*(*t*) = *pB*(*t*)/*c*. Moreover, since Eqs (1) and (4) are linear differential equations, *M*_1_(*t*) = *D*_1_*e*^-*dt*^ for *t* ≥ *τ*_1_ and *M*_2_(*t*) = *D*_2_*e*^-*dt*^ for *t* ≥ *τ*_2_: otherwise *M*_1_(t) = *M*_2_(*t*) = 0, respectively. Thus, the above Eqs (1–6) are further simplified assuming *τ*_1_ = 0 and *D*_1_ > 0, and we obtained the following single ordinary differential equation, which we used to analyze the antibody responses (i.e., IgG(S) titers (AU/mL)) in this study:

$$\frac{dA(t)}{dt}=H(t)\frac{{\left(D_{1}e^{-dt}+D_{2}e^{-d\left(t-\tau_{2}\right)}\right)}^{m}}{K^{m}+{\left(D_{1}e^{-dt}+D_{2}e^{-d\left(t-\tau_{2}\right)}\right)}^{m}}-\mu A(t),$$ (7)

Where *H*(*t*) = *H_i_* = *pP_i_*/c for *τ_i_* + *η_i_* ≤ *t<τ*_*i*+1_ + *η*_*i*+1_ (*i* = 1 or 2), *η*_3_ = ∞ and *D*_2_ > 0 for *τ*_2_ + *η*_2_ ≤ *t*: otherwise *D*_2_ = 0. This simple model can quantify the vaccine-elicited time-course antibody dynamics as described in **Fig 2A** under an arbitrary threshold of antibody titers *A*_TH_ (see below).

### Quantifying vaccine-elicited time-course antibody dynamics

In addition to the participants in the cohort, we included 12 health care workers whose serum was sequentially sampled for 40 days (on average 25 samples per individual) for validation and parameterization of a mathematical model for vaccine-elicited antibody dynamics. A nonlinear mixed effects model was used to fit the antibody dynamics model, given by Eq (7), to the longitudinal antibody titers of IgG(S) obtained from the 12 health care workers. The mathematical model included both a fixed effect and a random effect in each parameter. That is, the parameters for individual *k*, *θ_k_*(= *θ*×*e^π_k_^*) are represented as a product of *θ* (a fixed effect) and *e^π_k_^* (a random effect). *π*_*k*_ follows a normal distribution with mean 0 and standard deviation Ω. As we described above, since *η*_1_ > *η*_2_ and *P*_1_ < *P*_2_, we assumed *η*_1_ = *η* and *η*_2_ = *f*_delay_*η*, *H*_2_ = *H* and *H*_1_ = *f*_degree_*H*, and estimated *η*, *H*, 0 < *f*_delay_ < 1 and 0 < *f*_degree_ < 1 for conducting biologically reasonable estimations. We here assumed that the parameters *η*, *H*, *f*_delay_, *f*_degree_ and *m* varied across individuals, whereas we did not consider interindividual variability in other parameters to ensure parameter identifiability. Note that the half-life of mRNA (i.e., log 2/*d*) and dose of mRNA (i.e., *D*_*i*_) are assumed to be 1 day [64] and 100 (*μg*/0.5 mL) [65], respectively. Fixed effect and random effect were estimated by using the stochastic approximation expectation-approximation algorithm and empirical Bayes’ method, respectively. Fitting was performed using MONOLIX 2019R2 (www.lixoft.com) [66]. The estimated (fixed and individual) parameters are listed in **S1 Table**. With the estimated parameters for each individual, the dynamics of IgG(S) titers, *A*(*t*), and the average *de novo* antibody response elicited by the first and second vaccinations, $H(t){\left(D_{1}e^{-dt}+D_{2}e^{-d\left(t-\tau_{2}\right)}\right)}^{m}/\left(K^{m}+{\left(D_{1}e^{-dt}+D_{2}e^{-d\left(t-\tau_{2}\right)}\right)}^{m}\right)$, were calculated in **S5 Fig AB**, respectively. Interestingly, we observed that the variations induced by the second vaccination were much larger than those induced by the first vaccination.

We found that most of the best-fitted estimated parameters in the mathematical model (i.e., *D*_1_, *D*_2_, *d*, *μ*, *K*, *η*, *f*_delay_, *f*_degree_) were the same or similar across the 12 individuals compared with those of parameters of *m* and *H* (see **S1 Table**). We note that *m* and *H*, which showed wide variation of estimated values, contributed mainly to the vaccine-elicited antibody dynamics after the second vaccine dose, whereas the other parameters contributed to that after the first dose. In fact, we are interested in the large variation after the second dose (but not the negligible variation after the first dose). Therefore, we hereafter fixed the parameters in our mathematical model to be the estimated population parameters listed in **S1 Table**, except *m* and *H*. These assumptions enabled us to accurately reconstruct the large variations in antibody dynamics after the second dose, and these two parameters were independently estimated from each IgG(S) by a nonlinear least-squares method, even if 2,407 participants had only 2 or 3 measurements of antibody titers at different time points. Using the estimated parameters for each individual, we fully reconstructed the dynamics of IgG(S) titers of all participants after the first vaccination in **S5 Fig C**. We summarized the distribution of parameter values *m* and *H* for 2,407 participants in **S5 Fig D**, and the best-fit antibody titer curves of 200 randomly selected individuals are plotted along with the observed data for visualization in **S2 Fig**.

### Mathematical model validation

Outside the study period described in **Fig 1E**, to validate our mathematical model (i.e., Eqs (4–5)), we first prepared **Validation dataset A**: the additional sequentially performed serum sampling of 12 health care workers, that is, data for 3 to 4 timepoints before the booster dose (on average 4 additional samples per individual for 165 days after the first vaccine dose). Employing our estimated parameters, which were estimated from the original dataset (i.e., the black circles in **S6 Fig**), listed in **S1 Table**, we found our mathematical model predicted the values for the additional 3 to 4 timepoints before booster vaccination with high accuracy. Next, to validate the reconstructed best-fit antibody titer curves described in **S5 Fig C**, we also prepared **Validation dataset B**: an additional third or fourth antibody measurement from 110 of the 2407 participants of our Fukushima vaccination cohort around 3 to 4 months after the second or third timepoint data but before the booster vaccination. We compared the reconstructed antibody titer curves and the additional data and confirmed that the additional data points obtained from participants who were not infected with COVID-19 (i.e., IgG(N)-negative participants) during that period perfectly matched the prediction of our mathematical model (**S7 Fig**). On the other hand, interestingly, for those who got infected with COVID-19, the additional data were significantly larger than the reconstructed curves (**S8 Fig**).

Additionally, we assessed the accuracy of the reconstructed best-fit antibody titers by calculating the Pearson’s correlation coefficient between the reconstructed antibody titers and the observed (or additionally observed) antibody titers (**S9 Fig**). The Pearson’s correlation coefficients between the observed antibody titers, which were used in parameter estimation, and the reconstructed antibody titers, were 0.99 for both of the 12 health care workers and the 2407 participants in the Fukushima vaccination cohort. Furthermore, the Pearson’s correlation coefficients between the additionally observed antibody titers, which were not used in parameter estimation, and the reconstructed antibody tiers, were 0.94 and 0.92 for the 12 health care workers and the 110 participants in the Fukushima vaccination cohort, respectively.

Taken together, our additional data (i.e., **Validation dataset A** and **B**) and quantitative analysis demonstrated that our mathematical model and the reconstructed best-fit antibody titer curves using the same are validated for the purposes of predicting antibody dynamics in a generalized population.

### Building optimized antibody scores

A Python implementation of Ustun et al.’s [29] algorithm (risk-slim, https://github.com/ustunb/risk-slim) was used to build optimized AUC scores. Briefly, the algorithm searches for the best linear combination of features with integer coefficients that minimizes the sum of the logistic loss and the *l*_0_-norm of the coefficients. The range of coefficients was set to -5 to +5; the *l*_0_-penalty parameter C_0_ was set to 1×10^−3^.

### Evaluation of antibody scores

When an individual’s top (or bottom) AUC score is above a threshold, that individual is predicted to be in the top 1/3 (or the bottom 1/3) of the population. The accuracy, precision, and recall of the score are defined as (TP + TN)/(TP + TN + FP + FN), TP/(TP + FP), and TP/(TP + FN), respectively, where TP, TN, FP, and FN denote true positives (i.e., the number of individuals predicted to be positive that were actually positive), true negatives, false positives, and false negatives, respectively.

### Statistical analysis

The answers from the 2,407 participants who completed the paper-based questionnaire were converted into a set of categorical and numerical variables. Numerical variables included age, BMI, and the interval between the two doses. These variables were then used in a multiple regression analysis to explain the three antibody dynamics features. Six participants who did not fully answer the questionnaire were excluded from the analysis. The variables used here belonged to any of the five categories: (i) basic demographic information and lifestyle habits, (ii) information on vaccinations, (iii) underlying medical conditions, (iv) adverse reactions, and (v) medications being taken. When necessary, the same variables were compared among different generations or different groups using Pearson’s chi-square test (for categorical variables), analysis of variance (ANOVA, for more than two numerical variables), or Welch T-test (for two numerical variables). A Bonferroni correction was applied for multiple comparisons. All statistical analyses were performed using R (version 4.1.2) or JMP Pro 16.

### Map of Japan

The basemap shapefile of Japan and Fukushima Prefecture was downloaded from National Land Numerical Information (https://nlftp.mlit.go.jp/ksj/gml/datalist/KsjTmplt-N03-v2_4.html) and edited using the R package sf.

## Supporting information

S1 Fig Comparison between IgG(S) and neutralization activity. (DOCX)

S2 Fig Reconstructed antibody titer trajectory for individual participants. (DOCX)

S3 Fig Analyzing antibody titers. (DOCX)

S4 Fig Modeling vaccine-elicited B cell dynamics. (DOCX)

S5 Fig Calibrating vaccine-elicited antibody dynamics. (DOCX)

S6 Fig Validating calculated lgG(S) titers for each individual health care worker. (DOCX)

S7 Fig Validating reconstructed lgG(S) titers for 109 participants from the Fukushima vaccination cohorts who did not receive the booster vaccination and did not get infected with COVID-19. (DOCX)

S8 Fig Validating reconstructed lgG(S) titers for 10 participants from the Fukushima vaccination cohorts who did not receive the booster vaccination and got infected with COVID-19. (DOCX)

S9 Fig Evaluating estimation accuracy by mathematical model through a comparison of observed and estimated antibody titers. (DOCX)

S10 Fig Middle AUC scores. (DOCX)

S11 Fig Scoring for groups 1 and 4. (DOCX)

S1 Table Estimated fixed and individual parameters for 12 health care workers. (DOCX)

S2 Table Coefficients and p-values of the multiple regression analysis. (DOCX)

S3 Table Distributions of the top, bottom and middle AUC scores. (DOCX)

S1 Data Synthetic patient dataset. (CSV)
