# Supplementary material for: Modeling and predicting individual variation in COVID-19 vaccine-elicited antibody response in the general population
Source: PLOS Digit Health. 2024 May 3;3(5):e0000497. doi: 10.1371/journal.pdig.0000497 (PMC11068210; doi:10.1371/journal.pdig.0000497)
Supplement: S1 Fig — (DOCX) [file pdig.0000497.s001.docx]

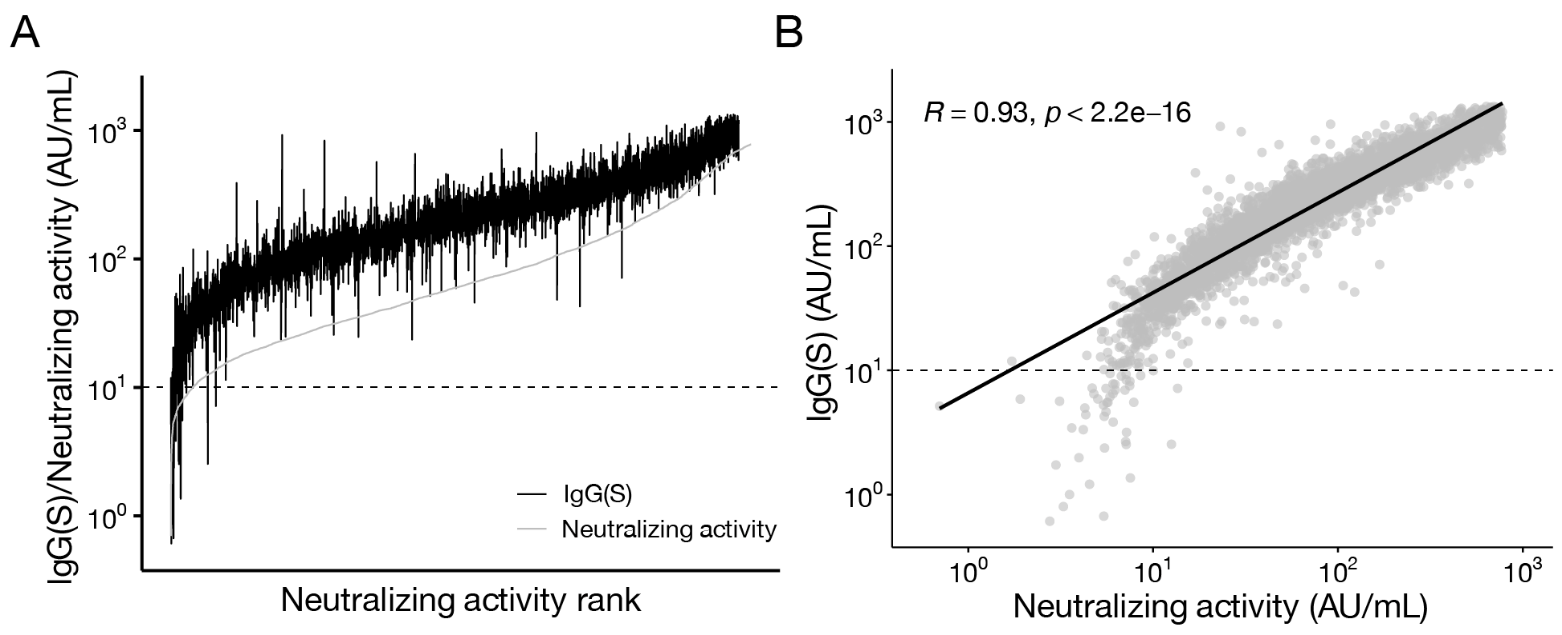
**Supplementary Figure 1.** **Comparison between IgG(S) and** **neutralization activity: (A)** Curves with IgG(S) and neutralization activity on the *Y*-axis and its corresponding neutralization activity rank on the *X*-axis are plotted in black and gray, respectively. **(B)** Correlations between IgG(S) and neutralization activity from same samples are described. Data points represent individual samples. Correlations were calculated as Pearson correlation coefficients.
