# Supplementary material for: Modeling and predicting individual variation in COVID-19 vaccine-elicited antibody response in the general population
Source: PLOS Digit Health. 2024 May 3;3(5):e0000497. doi: 10.1371/journal.pdig.0000497 (PMC11068210; doi:10.1371/journal.pdig.0000497)
Supplement: S2 Fig — (DOCX) [file pdig.0000497.s002.docx]

**Supplementary Figure 2. Reconstructed antibody titer trajectory for individual participants:** The estimated antibody titer for each individual participant (solid lines) along with the observed data (closed dots) are depicted using the best-fit parameter estimates. The curve of 200 was randomly selected for visualization because of the large number.
