# Supplementary material for: Modeling and predicting individual variation in COVID-19 vaccine-elicited antibody response in the general population
Source: PLOS Digit Health. 2024 May 3;3(5):e0000497. doi: 10.1371/journal.pdig.0000497 (PMC11068210; doi:10.1371/journal.pdig.0000497)
Supplement: S3 Fig — (DOCX) [file pdig.0000497.s003.docx]

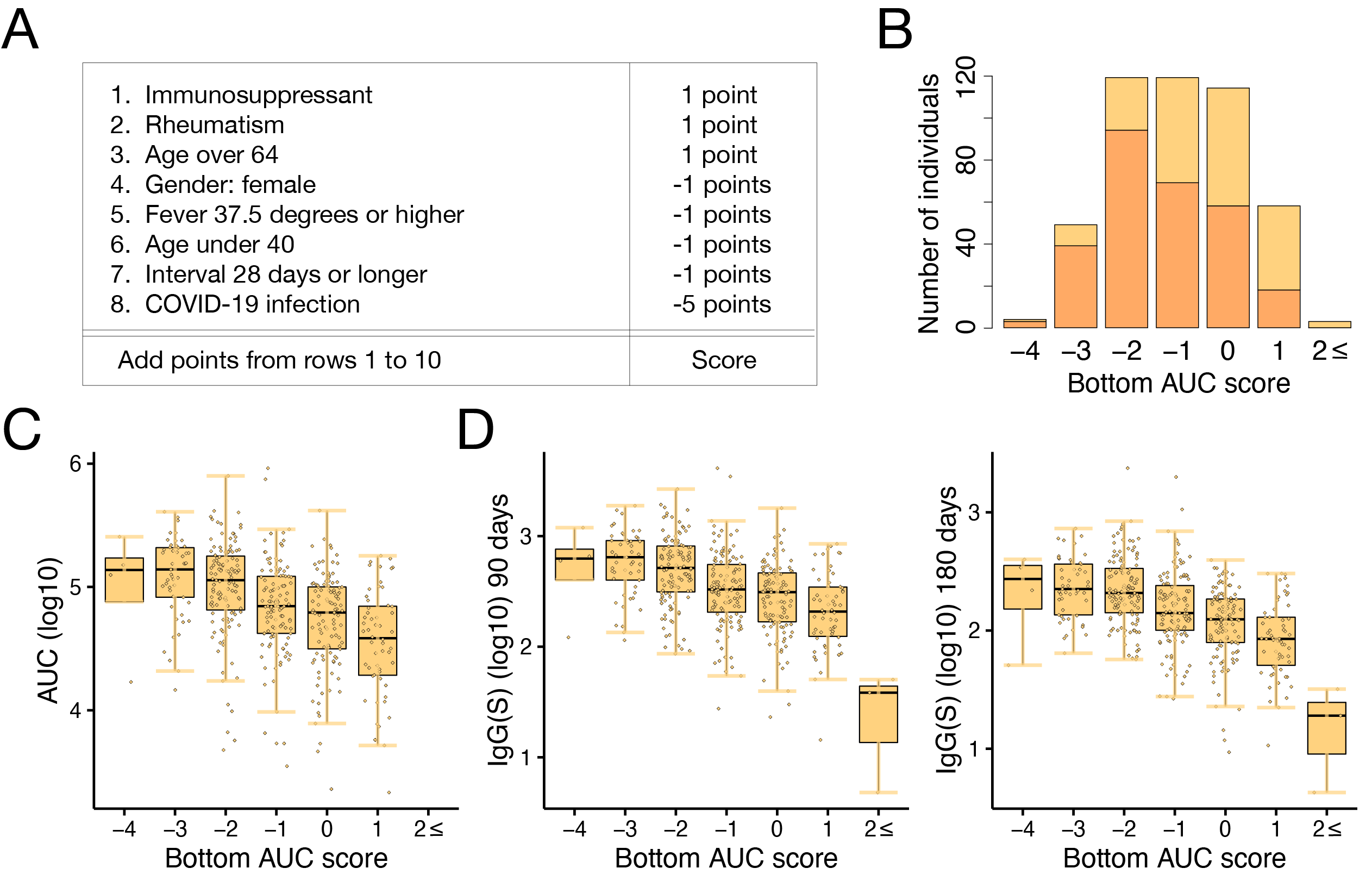
**Supplementary Figure 3.** **Analyzing antibody titers: (A)** The bottom AUC score to identify individuals with AUC in the bottom third of the population is shown. **(B)** The distribution of the bottom AUC score in the test dataset. 4, 49, 119, 119, 114, 58, and 3 individuals had scores of -4, -3, -2, -1, 0, 1, or 2 or more, respectively. Those in the bottom third of the test dataset are shown in yellow, and those not in the bottom third are shown in orange. The ratio of individuals with AUC in the bottom third of the test dataset increased as the bottom AUC score increased. **(C)** The average AUC tended to decrease as the bottom AUC score increased. The 3 individuals with a score of 2 had an AUC of 0, which is not shown in the plot. **(D)** The bottom AUC score was inversely correlated with the IgG(S) titers 90 or 180 days from the second vaccination, calculated from the mathematical model.
