# Supplementary material for: Modeling and predicting individual variation in COVID-19 vaccine-elicited antibody response in the general population
Source: PLOS Digit Health. 2024 May 3;3(5):e0000497. doi: 10.1371/journal.pdig.0000497 (PMC11068210; doi:10.1371/journal.pdig.0000497)
Supplement: S4 Fig — (DOCX) [file pdig.0000497.s004.docx]

**
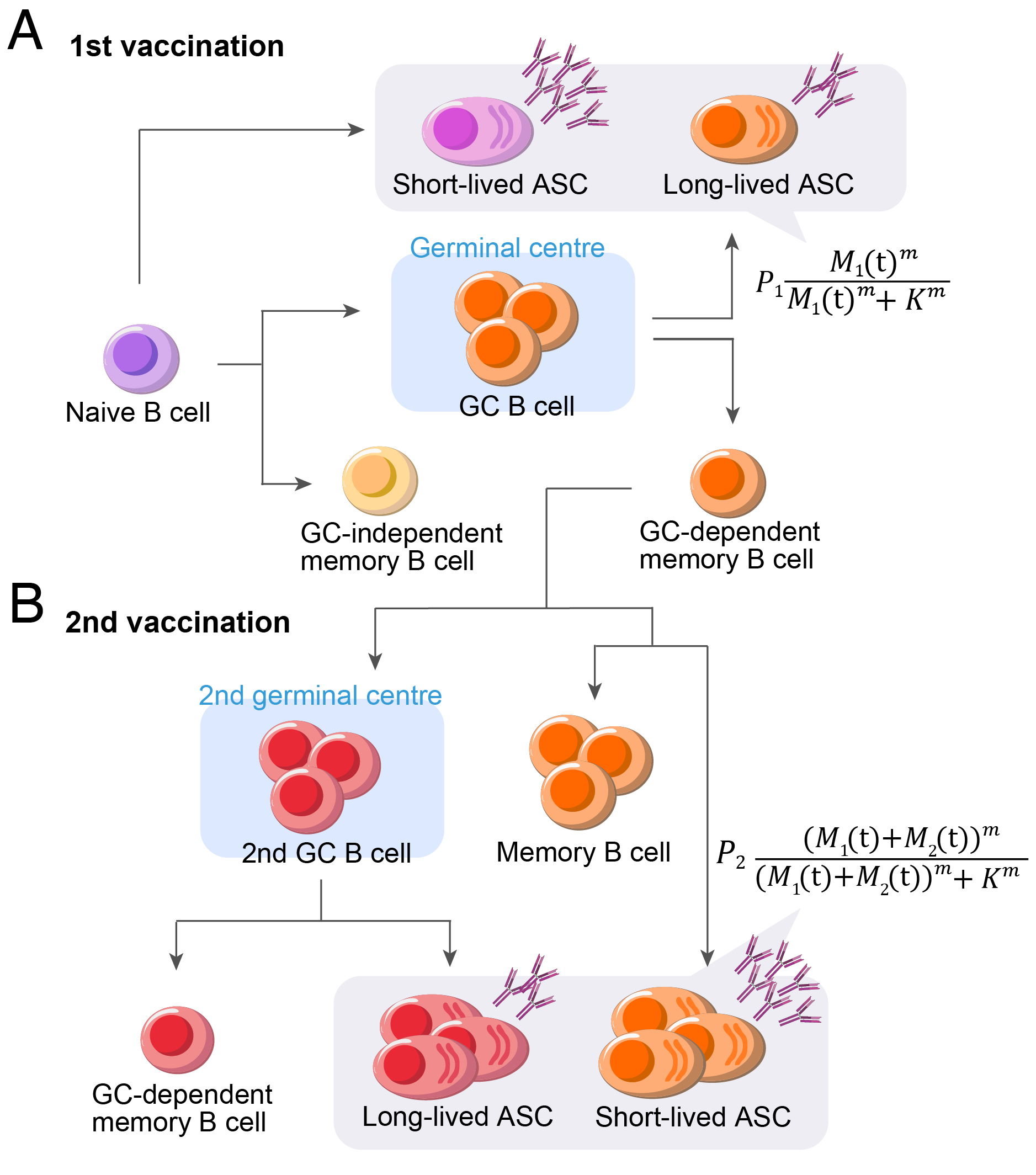
Supplementary Figure 4.** **Modeling vaccine-elicited B cell dynamics:** **(A)** First vaccination-elicited antibody-secreting cell and memory B cell inductions are described. Once naïve B cells encounter vaccine antigens outside the germinal center (GC), the activated naïve B cells differentiate into short-lived antibody-secreting cells (ASCs), plasmablasts, GC B cells, or GC-independent memory B cells depending on BCR affinity for their cognate antigen. Subsequently, the GC B cells undergo rapid proliferation with somatic immunoglobulin hypermutation and differentiate into GC-dependent memory B cells or long-lived antibody-secreting cells (plasma cells), with immunoglobulin class switching. Through the GC-independent and dependent pathways, antibody-secreting cells (i.e., $B(t)$) are induced and they secrete antibodies (i.e., $A(t)$). **(B)** Second, vaccination-elicited recall immune responses are described. After re-exposure to vaccine antigens, memory B cells rapidly reactivate and expand. Of activated memory B cells, while some differentiate into plasmablasts or memory B cells outside the GC, others enter the GC to be secondary GC B cells. These secondary GC B cells differentiate into GC-dependent memory B cells or plasma cells. In general, the secondary antibody responses are much faster and larger by an order of magnitude compared with the first vaccination-elicited antibody responses.
