# Supplementary material for: Modeling and predicting individual variation in COVID-19 vaccine-elicited antibody response in the general population
Source: PLOS Digit Health. 2024 May 3;3(5):e0000497. doi: 10.1371/journal.pdig.0000497 (PMC11068210; doi:10.1371/journal.pdig.0000497)
Supplement: S5 Fig — (DOCX) [file pdig.0000497.s005.docx]

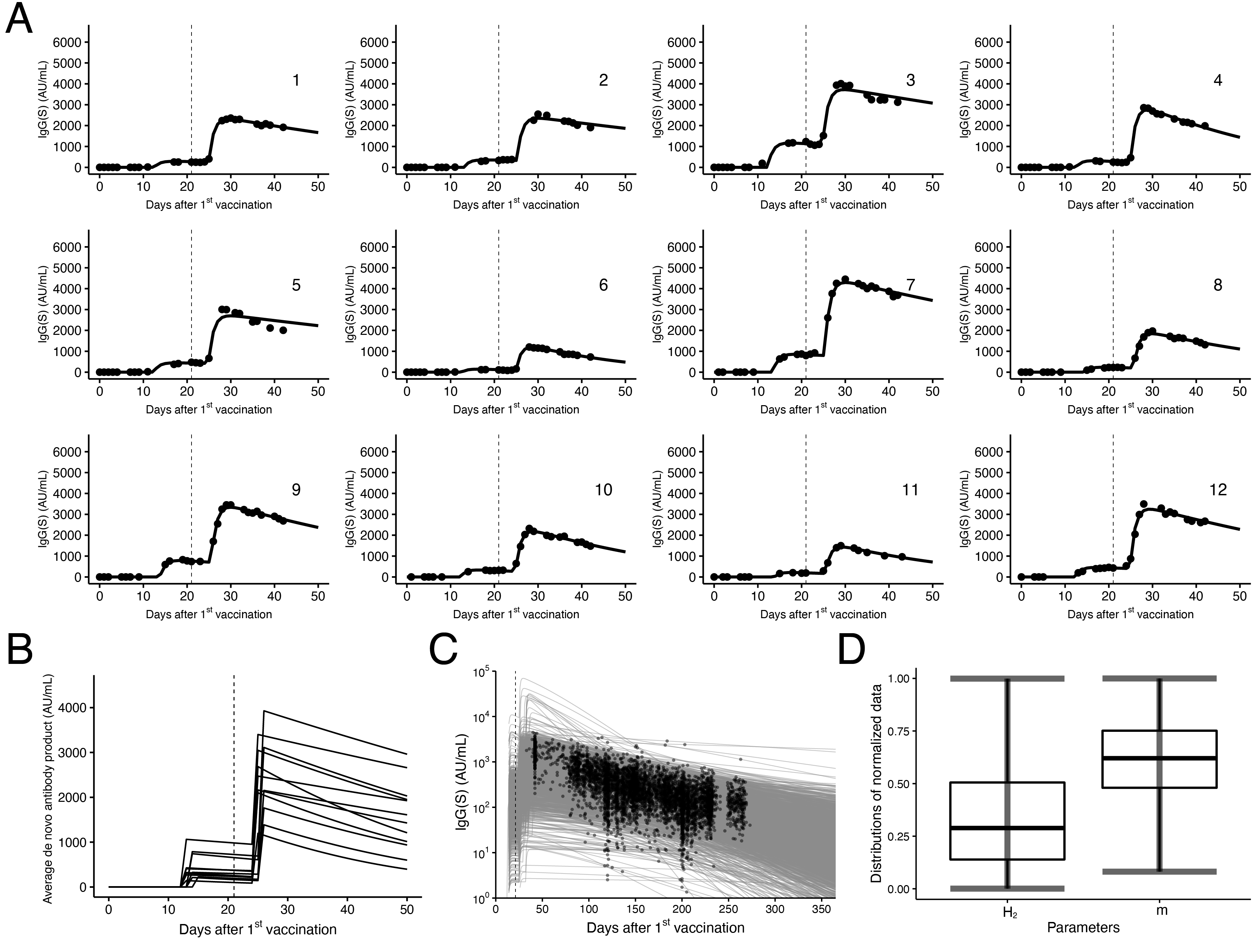
**Supplementary Figure 5.** **Calibrating vaccine-elicited antibody dynamics: (A)** Observed and best-fitted IgG(S) titers are described for the 12 health care workers (HCWs) whose serum was sequentially sampled. The dashed vertical lines at day 21 correspond to the date of second vaccination. **(B)** Time-course averages of *de novo* antibody response elicited by the first and second vaccinations for the 12 HCWs are described. **(C)** Reconstructed individual antibody dynamics for the 2,159 participants are represented along with the measured IgG(S). The black circles correspond to the measurements of antibody titers at different time points. **(D)** Distributions of the estimated parameter values (i.e., $H_{2}$ and $m$) for 2,407 participants are plotted. Dataset for each distribution is normalized by the value corresponding to the 95th percentile of data values, and data whose values were larger than this value were removed to improve visibility of the figure.
