# Supplementary material for: Modeling and predicting individual variation in COVID-19 vaccine-elicited antibody response in the general population
Source: PLOS Digit Health. 2024 May 3;3(5):e0000497. doi: 10.1371/journal.pdig.0000497 (PMC11068210; doi:10.1371/journal.pdig.0000497)
Supplement: S6 Fig — (DOCX) [file pdig.0000497.s006.docx]

**
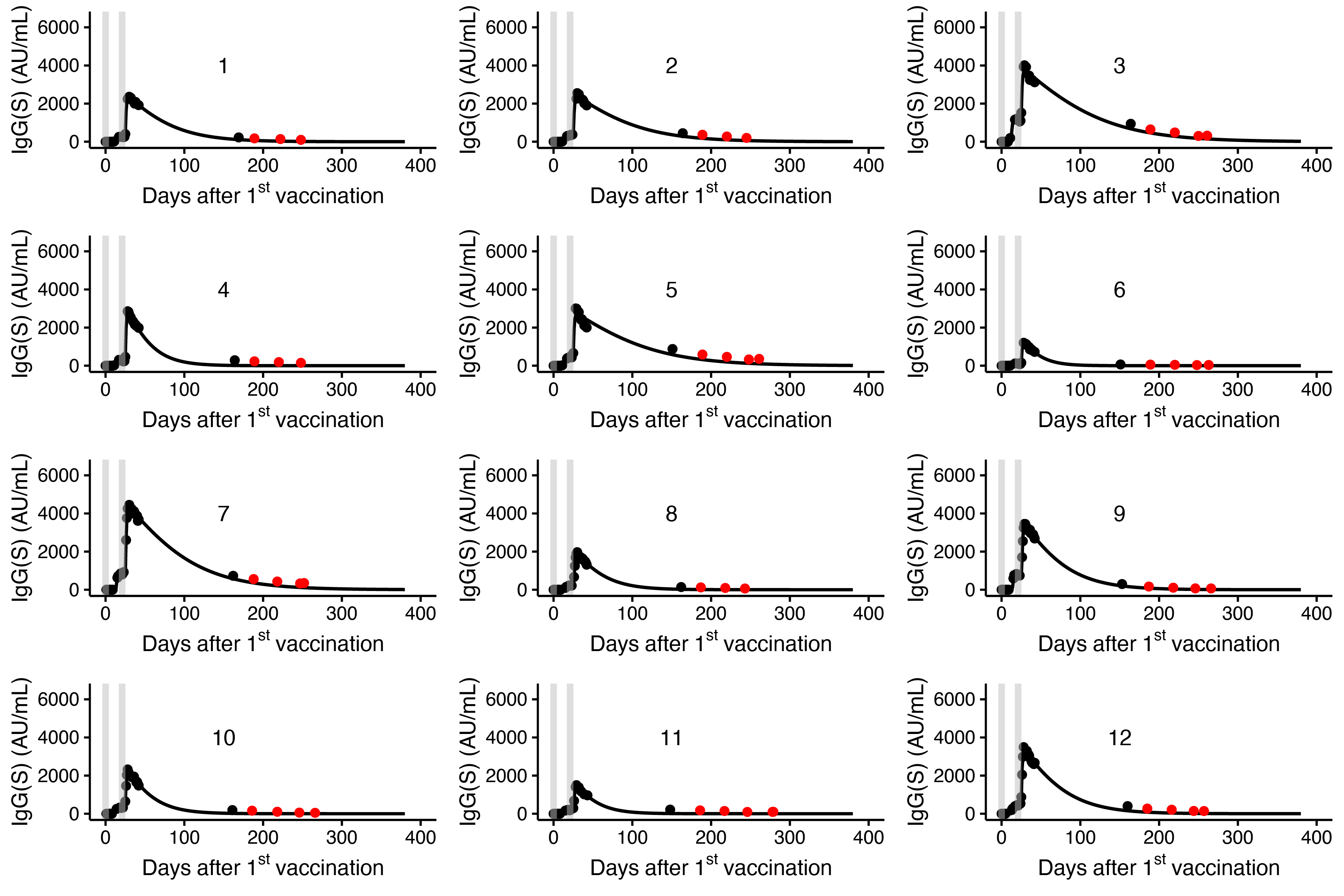
Supplementary Figure 6.** **Validating calculated lgG(S) titers for each individual health care worker:** Observed and calculated lgG(S) titers for each individual health care worker before the booster vaccination: The black circles correspond to the observed lgG(S) titers described in **S5 Fig A**, and the red circles correspond to the additionally observed lgG(S) titers before the booster vaccination (i.e., **Validation dataset A**). The gray shaded vertical lines correspond to the dates of the 1st and 2nd vaccination. The solid curves describe the lgG(S) titer calculated by use of our mathematical model with the previously estimated parameters listed in **S1 Table**.
