# Supplementary material for: Modeling and predicting individual variation in COVID-19 vaccine-elicited antibody response in the general population
Source: PLOS Digit Health. 2024 May 3;3(5):e0000497. doi: 10.1371/journal.pdig.0000497 (PMC11068210; doi:10.1371/journal.pdig.0000497)
Supplement: S8 Fig — (DOCX) [file pdig.0000497.s008.docx]

**
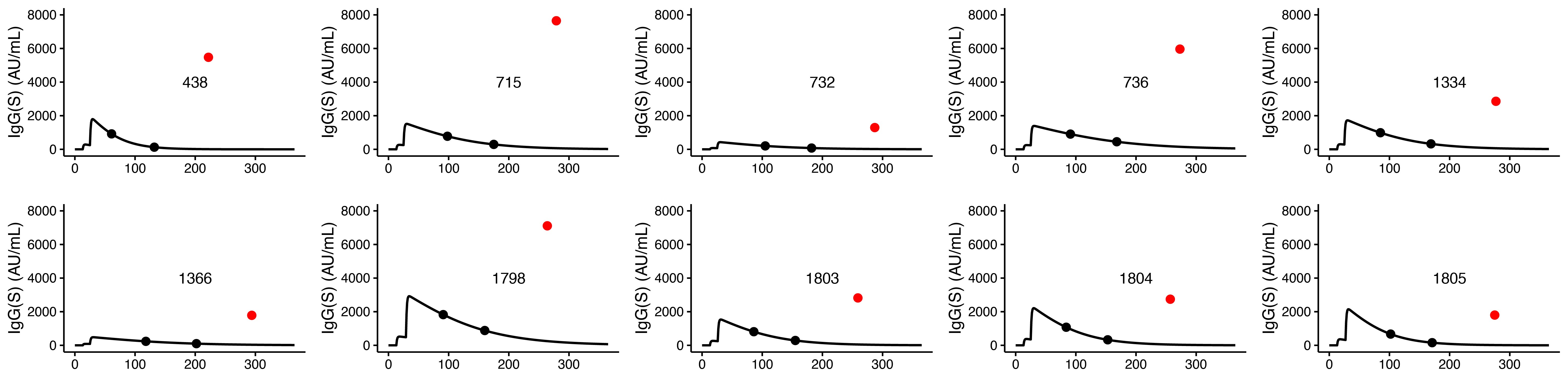
Supplementary Figure 8.** **Validating reconstructed lgG(S) titers for 10 participants from the Fukushima vaccination cohorts who did not receive the booster vaccination and got infected with COVID-19:** The black circles correspond to the observed lgG(S) titers described in **S5 Fig C**, and the red circles correspond to the additionally observed lgG(S) titers before the booster vaccination (i.e., IgG(N)-positive participants in **Validation dataset B**), respectively. The curves describe the reconstructed best-fit antibody titer curves described in **S5 Fig C**.
