# Supplementary material for: Modeling and predicting individual variation in COVID-19 vaccine-elicited antibody response in the general population
Source: PLOS Digit Health. 2024 May 3;3(5):e0000497. doi: 10.1371/journal.pdig.0000497 (PMC11068210; doi:10.1371/journal.pdig.0000497)
Supplement: S9 Fig — (DOCX) [file pdig.0000497.s009.docx]

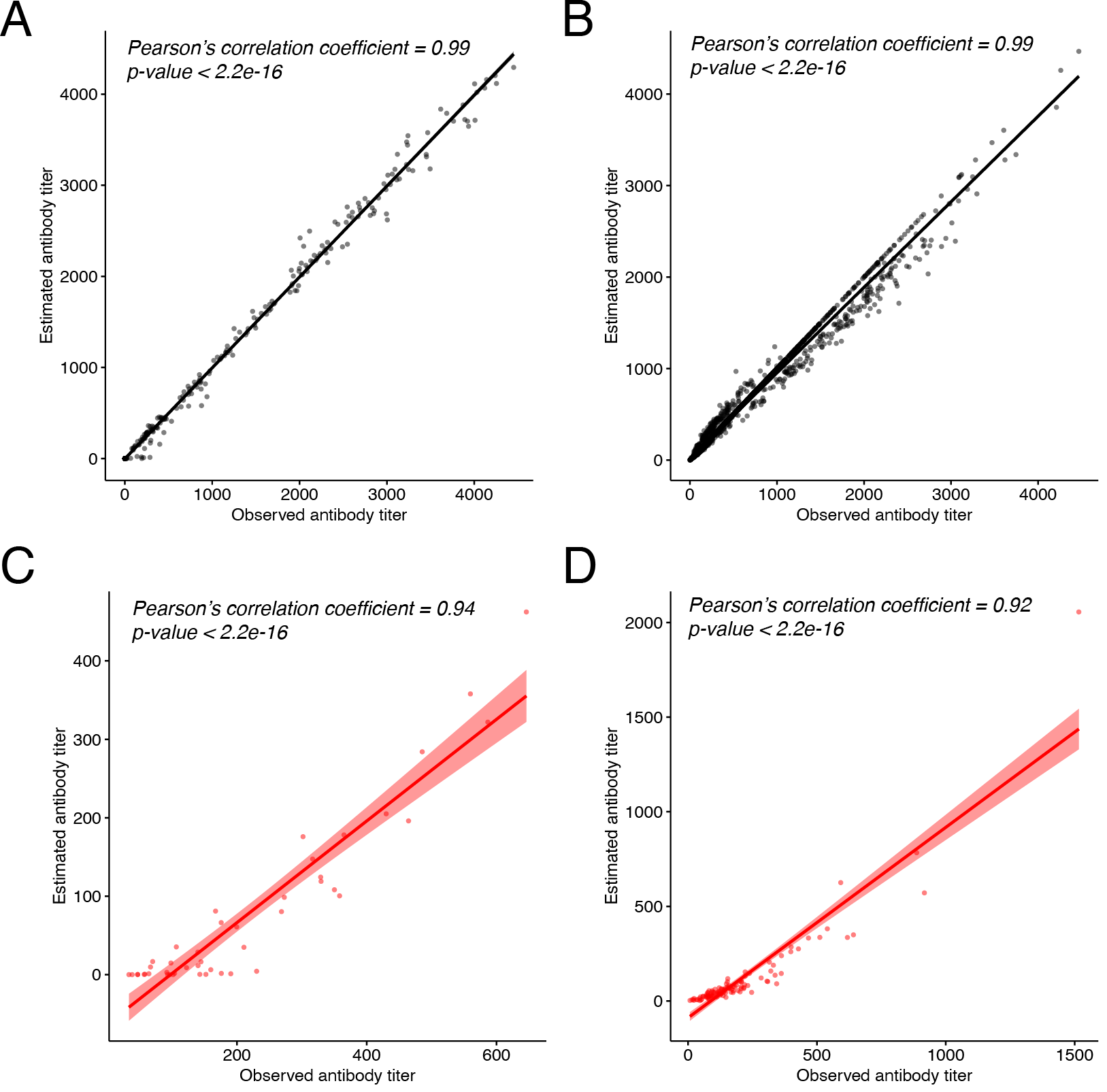


**Supplementary Figure 9.** **Evaluating estimation accuracy by mathematical model through a comparison of observed and estimated antibody titers: (A)** and **(B)** show the correlation between the observed IgG(S) and the estimated IgG(S) for **(A)** 12 health care workers (**S5 Fig A**), and **(B)** 2407 participants in the Fukushima vaccination cohort (**S2 Fig**), respectively. **(C)** and **(D)** show the correlation between the additionally observed IgG(S) (i.e., observed data which are not used in the parameter estimation) and the estimated IgG(S) for **(C)** 12 health care workers (**S6 Fig** and **Validation dataset A**), and **(D)** 110 participants in Fukushima vaccination cohort (**S7 Fig** and **Validation dataset B**), respectively. The Pearson’s correlation coefficients and their p-values are described in the top of panel for each plot.
