# Supplementary material for: Modeling and predicting individual variation in COVID-19 vaccine-elicited antibody response in the general population
Source: PLOS Digit Health. 2024 May 3;3(5):e0000497. doi: 10.1371/journal.pdig.0000497 (PMC11068210; doi:10.1371/journal.pdig.0000497)
Supplement: S10 Fig — (DOCX) [file pdig.0000497.s010.docx]

| 1. Collagen disease | 3 points |
| --- | --- |
| 2. Interval 28 days or longer | 1 point |
| 3. Steroids | -2 points |
| Add points from rows 1 to 3 | Score |

**Supplementary Figure 10**. **Middle AUC score**
