# Supplementary material for: Modeling and predicting individual variation in COVID-19 vaccine-elicited antibody response in the general population
Source: PLOS Digit Health. 2024 May 3;3(5):e0000497. doi: 10.1371/journal.pdig.0000497 (PMC11068210; doi:10.1371/journal.pdig.0000497)
Supplement: S11 Fig — (DOCX) [file pdig.0000497.s011.docx]

**
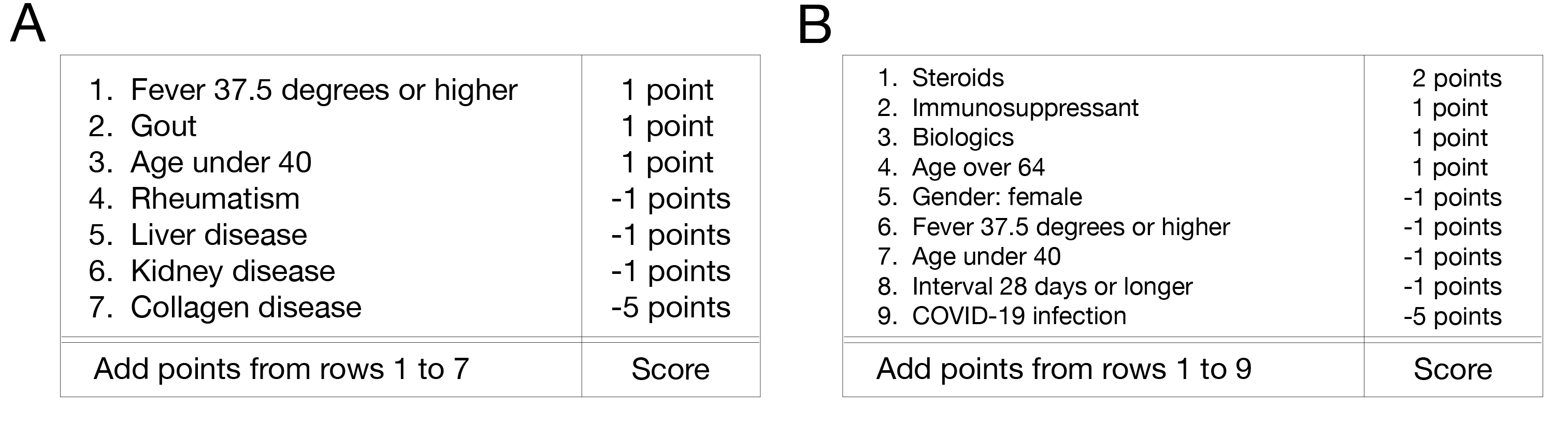
**

**Supplementary Figure 11**. **Scoring for groups 1 and 4:** **(A)** Group 1 score to identify individuals in Group 1 is shown. **(B)** Group 4 score to identify individuals in Group 4 is shown.
