# Supplementary material for: Modeling and predicting individual variation in COVID-19 vaccine-elicited antibody response in the general population
Source: PLOS Digit Health. 2024 May 3;3(5):e0000497. doi: 10.1371/journal.pdig.0000497 (PMC11068210; doi:10.1371/journal.pdig.0000497)
Supplement: S1 Table — (DOCX) [file pdig.0000497.s012.docx]

**Supplementary Table 1**. Estimated fixed and individual parameters for 12 health care workers

| **Parameter** **or****variable** | Decay rate ofantibody-secreting cells | Maximum *de novo* production of antibody by 1^st^ vaccination | | Delay of induction antibody-secreting cellsafter1^st^ vaccination | | Steepness at which induction increases with increasing the amount of mRNA | Amount of mRNA satisfying $P_{i}/2$ | Maximum *de novo* production of antibody by 2^nd^ vaccination | Delay of induction antibody-secreting cellsafter2^nd^ vaccination |
| --- | --- | --- | --- | --- | --- | --- | --- | --- | --- |
| **Symbol** | $\mu$ | $H_{1}$ | $\eta_{1}$ | | $m$ | | $K$ | $H_{2}$ | $\eta_{2}$ |
| **Unit** | day^-1^ | AU/mL | day | | --- | | $\mu g/0.5\mathrm{mL}$ | AU/mL | day |
| **Individual estimated parameters for** $\boldsymbol{S}\boldsymbol{1}$ **to** $\boldsymbol{S}\boldsymbol{12}$ | | | | | | | | | |
| $S1$ | $0.875222$ | $733.267$ | $12.5456$ | | $0.0368328$ | | $28100$ | $5110.48$ | $3.9297$ |
| $S2$ | $0.875222$ | $830.423$ | $13.0646$ | | $0.0282303$ | | $28100$ | $4938.44$ | $4.1248$ |
| $S3$ | $0.875222$ | $2566.454$ | $12.2374$ | | $0.0236777$ | | $28100$ | $7551.46$ | $3.8062$ |
| $S4$ | $0.875222$ | $1076.899$ | $12.5519$ | | $0.0623210$ | | $28100$ | $7221.46$ | $3.9135$ |
| $S5$ | $0.875222$ | $986.460$ | $12.3896$ | | $0.0240698$ | | $28100$ | $5500.07$ | $3.8658$ |
| $S6$ | $0.875222$ | $610.840$ | $12.6080$ | | $0.0807321$ | | $28100$ | $3560.90$ | $3.9554$ |
| $S7$ | $0.875222$ | $1988.887$ | $13.4408$ | | $0.0275337$ | | $28100$ | $8976.42$ | $4.2119$ |
| $S8$ | $0.875222$ | $756.918$ | $14.5003$ | | $0.0532348$ | | $28100$ | $4624.04$ | $4.6832$ |
| $S9$ | $0.875222$ | $2091.734$ | $13.7844$ | | $0.0388568$ | | $28100$ | $7544.93$ | $4.5142$ |
| $S10$ | $0.875222$ | $1076.296$ | $12.3075$ | | $0.0580142$ | | $28100$ | $5520.77$ | $3.8273$ |
| $S11$ | $0.875222$ | $802.460$ | $13.8106$ | | $0.0667276$ | | $28100$ | $3932.26$ | $4.4253$ |
| $S12$ | $0.875222$ | $1185.324$ | $12.4518$ | | $0.0390830$ | | $28100$ | $7282.56$ | $3.8582$ |
| **Population estimated parameters** | | | | | | | | | |
| --- | $0.875222$ | $1097.69$ | $13.01$ | | $0.0423437$ | | $28100$ | $5786.453$ | $4.11$ |
