# Supplementary material for: Modeling and predicting individual variation in COVID-19 vaccine-elicited antibody response in the general population
Source: PLOS Digit Health. 2024 May 3;3(5):e0000497. doi: 10.1371/journal.pdig.0000497 (PMC11068210; doi:10.1371/journal.pdig.0000497)
Supplement: S2 Table — (DOCX) [file pdig.0000497.s013.docx]

**Supplementary Table 2**. Coefficients and p-values in the multiple regression analysis

|  | log of Peak | | | | Duration | | | | log of AUC | | | |
| --- | --- | --- | --- | --- | --- | --- | --- | --- | --- | --- | --- | --- |
|  | **B*** | **SE*** | **β*** | **p*** | **B** | **SE** | **β** | **p** | **B** | **SE** | **β** | **p** |
| Age | -0.0074 | 0.0007 | -0.3242 | **<0.0001** | -1.5629 | 0.1245 | -0.3530 | **<0.0001** | -0.0159 | 0.0016 | -0.2967 | **<0.0001** |
| Interval | 0.0177 | 0.0024 | 0.1513 | **<0.0001** | 0.8693 | 0.4522 | 0.0380 | 0.0547 | 0.0250 | 0.0057 | 0.0902 | **<0.0001** |
| BMI | 0.0011 | 0.0022 | 0.0110 | 0.6056 | 0.6008 | 0.4081 | 0.0303 | 0.1411 | 0.0111 | 0.0051 | 0.0460 | 0.0303 |
| Gender: male | -0.0430 | 0.0096 | -0.1047 | **<0.0001** | -2.9094 | 1.8176 | -0.0363 | 0.1096 | -0.0527 | 0.0228 | -0.0542 | 0.0208 |
| Local pain | 0.0098 | 0.0089 | 0.0237 | 0.2707 | 2.2583 | 1.6723 | 0.0280 | 0.1771 | 0.0467 | 0.0210 | 0.0478 | 0.0260 |
| Fever <37.5 deg | 0.0331 | 0.0128 | 0.0592 | 0.0098 | 7.0289 | 2.4177 | 0.0643 | **0.0037** | 0.0723 | 0.0303 | 0.0545 | 0.0171 |
| Fever ≥37.5 deg | 0.0546 | 0.0115 | 0.1217 | **<0.0001** | 12.2469 | 2.1792 | 0.1399 | **<0.0001** | 0.0929 | 0.0273 | 0.0875 | **0.0007** |
| Fatigue | 0.0014 | 0.0100 | 0.0034 | 0.8891 | 3.0750 | 1.8811 | 0.0386 | 0.1023 | 0.0318 | 0.0236 | 0.0330 | 0.1771 |
| Headache | -0.0057 | 0.0108 | -0.0124 | 0.5997 | 0.2283 | 2.0458 | 0.0025 | 0.9112 | -0.0174 | 0.0256 | -0.0160 | 0.4974 |
| Joint pain | 0.0043 | 0.0097 | 0.0098 | 0.6552 | 2.2652 | 1.8335 | 0.0262 | 0.2168 | 0.0298 | 0.0230 | 0.0285 | 0.1948 |
| Diarrhea | 0.0006 | 0.0291 | 0.0004 | 0.9842 | 3.2004 | 5.4902 | 0.0118 | 0.5600 | 0.0091 | 0.0688 | 0.0028 | 0.8953 |
| Nausea | 0.0281 | 0.0237 | 0.0257 | 0.2367 | 0.8825 | 4.4756 | 0.0041 | 0.8437 | 0.0176 | 0.0561 | 0.0068 | 0.7537 |
| Dizziness | -0.0123 | 0.0219 | -0.0120 | 0.5743 | -3.4944 | 4.1404 | -0.0174 | 0.3988 | -0.0553 | 0.0519 | -0.0227 | 0.2870 |
| Other symptoms | -0.0045 | 0.0189 | -0.0049 | 0.8106 | -2.6745 | 3.5645 | -0.0149 | 0.4532 | -0.0009 | 0.0447 | -0.0004 | 0.9833 |
| Smoking | -0.0201 | 0.0111 | -0.0388 | 0.0699 | -10.4631 | 2.0947 | -0.1031 | **<0.0001** | -0.0500 | 0.0262 | -0.0406 | 0.0571 |
| Drinking habit  Almost not  Occasionally  Every day | (0)  -0.0118  0.0140 | 0.0109  0.0147 | -0.0257  0.0260 | 0.2797  0.3423 | (0)  0.9136  2.0558 | 2.0579  2.7729 | 0.0102  0.0196 | 0.6571  0.4585 | (0)  0.0055  0.0472 | 0.0258  0.0347 | 0.0050  0.0371 | 0.8322  0.1746 |
| Daily alcohol intake  <20g  ≥20 & <40g  ≥40 & <60g  ≥60g | (0)  -0.0284  -0.0162  0.0147 | 0.0139  0.0224  0.0441 | -0.0526  -0.0162  0.0070 | 0.0412  0.4714  0.7383 | (0)  -6.9460  -9.3495  -11.6006 | 2.6248  4.2352  8.3129 | -0.0658  -0.0480  -0.0283 | **0.0082**  0.0274  0.1630 | (0)  -0.0277  -0.0574  -0.1116 | 0.0329  0.0531  0.1042 | -0.0217  -0.0243  -0.0225 | 0.3994  0.2797  0.2840 |
| Steroids | -0.0769 | 0.0346 | -0.0509 | 0.0264 | -21.8541 | 6.5325 | -0.0741 | **0.0008** | -0.3656 | 0.0819 | -0.1022 | **<0.0001** |
| NSAIDs | -0.0428 | 0.0172 | -0.0522 | 0.0127 | -7.7630 | 3.2408 | -0.0485 | 0.0167 | -0.0342 | 0.0406 | -0.0176 | 0.4002 |
| Acetaminophen | 0.0217 | 0.0283 | 0.0160 | 0.4431 | -3.4123 | 5.3332 | -0.0129 | 0.5224 | 0.0475 | 0.0668 | 0.0148 | 0.4773 |
| Antihistamine | -0.0002 | 0.0195 | -0.0002 | 0.9934 | -3.1805 | 3.6854 | -0.0180 | 0.3882 | -0.0180 | 0.0462 | -0.0084 | 0.6967 |
| Immunosuppressant | -0.1025 | 0.0495 | -0.0462 | 0.0384 | -27.1930 | 9.3357 | -0.0628 | **0.0036** | -0.5833 | 0.1170 | -0.1111 | **<0.0001** |
| Biologic agent | -0.0183 | 0.0613 | -0.0067 | 0.7651 | -7.8388 | 11.5614 | -0.0146 | 0.4978 | -0.2035 | 0.1449 | -0.0312 | 0.1603 |
| Anti-cancer agent | -0.0510 | 0.0684 | -0.0158 | 0.4560 | -16.9393 | 12.9131 | -0.0269 | 0.1897 | -0.4019 | 0.1618 | -0.0527 | 0.0131 |
| COVID-19 | 0.1940 | 0.1316 | 0.0301 | 0.1405 | 40.8721 | 24.8234 | 0.0325 | 0.0998 | 0.4587 | 0.3111 | 0.0301 | 0.1405 |
| Hypertension | 0.0215 | 0.0115 | 0.0459 | 0.0613 | 4.9383 | 2.1649 | 0.0541 | 0.0227 | 0.0463 | 0.0271 | 0.0418 | 0.0882 |
| Diabetes | -0.0334 | 0.0171 | -0.0418 | 0.0502 | -3.8822 | 3.2189 | -0.0248 | 0.2279 | -0.0090 | 0.0403 | -0.0048 | 0.8234 |
| Asthma | -0.0225 | 0.0213 | -0.0219 | 0.2914 | -5.1364 | 4.0167 | -0.0256 | 0.2011 | -0.1072 | 0.0503 | -0.0441 | 0.0332 |
| Anaphylaxis | -0.0020 | 0.0541 | -0.0008 | 0.9702 | 0.2621 | 10.2076 | 0.0005 | 0.9795 | -0.0973 | 0.1279 | -0.0156 | 0.4468 |
| Mental disease | 0.0216 | 0.0338 | 0.0131 | 0.5234 | -6.3821 | 6.3748 | -0.0198 | 0.3169 | 0.0363 | 0.0799 | 0.0093 | 0.6498 |
| COVID-19 (family) | 0.0406 | 0.0695 | 0.0118 | 0.5593 | -14.0813 | 13.1189 | -0.0209 | 0.2832 | 0.0260 | 0.1644 | 0.0032 | 0.8744 |
| Gout | 0.0354 | 0.0248 | 0.0299 | 0.1540 | 1.6894 | 4.6840 | 0.0073 | 0.7184 | 0.0900 | 0.0587 | 0.0321 | 0.1255 |
| Dyslipidemia | 0.0679 | 0.0136 | 0.1060 | **<0.0001** | 7.2468 | 2.5742 | 0.0579 | **0.0049** | 0.1148 | 0.0323 | 0.0757 | **0.0004** |
| Rheumatism | -0.0630 | 0.0399 | -0.0364 | 0.1148 | -16.5986 | 7.5334 | -0.0491 | 0.0277 | -0.2540 | 0.0944 | -0.0619 | **0.0072** |
| Lung disease | -0.0006 | 0.0291 | -0.0004 | 0.9830 | 0.2719 | 5.4931 | 0.0010 | 0.9605 | -0.0151 | 0.0688 | -0.0045 | 0.8267 |
| Heart disease | 0.0179 | 0.0170 | 0.0223 | 0.2920 | -1.7532 | 3.2102 | -0.0112 | 0.5850 | 0.0328 | 0.0402 | 0.0173 | 0.4149 |
| Collagen disease | -0.0638 | 0.0591 | -0.0232 | 0.2810 | -17.6793 | 11.1555 | -0.0329 | 0.1132 | 0.1119 | 0.1398 | 0.0172 | 0.4237 |
| Allergy | -0.0116 | 0.0172 | -0.0147 | 0.5018 | 4.1557 | 3.2506 | 0.0270 | 0.2013 | 0.0052 | 0.0407 | 0.0028 | 0.8980 |
| Immunodeficiency | 0.0748 | 0.1064 | 0.0142 | 0.4819 | 2.1067 | 20.0706 | 0.0021 | 0.9164 | 0.1402 | 0.2515 | 0.0113 | 0.5772 |
| Cancer | -0.0155 | 0.0256 | -0.0129 | 0.5455 | 5.5605 | 4.8328 | 0.0237 | 0.2500 | 0.0318 | 0.0606 | 0.0112 | 0.6000 |
| Thyroid disease | 0.0038 | 0.0274 | 0.0029 | 0.8888 | 12.0075 | 5.1685 | 0.0458 | 0.0203 | 0.2647 | 0.0648 | 0.0083 | 0.6834 |
| Liver disease | -0.0342 | 0.0202 | -0.0352 | 0.0907 | -8.1166 | 3.8103 | -0.0428 | 0.0333 | -0.0816 | 0.0477 | -0.0355 | 0.0876 |
| Kidney disease | -0.0169 | 0.0409 | -0.0085 | 0.6797 | -7.2212 | 7.7111 | -0.0185 | 0.3491 | -0.0265 | 0.0966 | -0.0560 | **0.0062** |

* B, SE, β, and p are partial regression coefficient, standard error, standardized partial regression coefficient, and p-value, respectively.
