# Supplementary material for: Modeling and predicting individual variation in COVID-19 vaccine-elicited antibody response in the general population
Source: PLOS Digit Health. 2024 May 3;3(5):e0000497. doi: 10.1371/journal.pdig.0000497 (PMC11068210; doi:10.1371/journal.pdig.0000497)
Supplement: S3 Table — (DOCX) [file pdig.0000497.s014.docx]

**Supplementary Table 3** | Distributions of the top, bottom and middle AUC scores.

| **Top AUC score** | **-6** | **-5** | **-1** | **0** | **1** | **2** | **3** | **4** |
| --- | --- | --- | --- | --- | --- | --- | --- | --- |
| Top 1/3 | 0 | 1 | 4 | 19 | 20 | 50 | 43 | 6 |
| Not in top 1/3 | 1 | 0 | 29 | 107 | 86 | 69 | 29 | 2 |

| **Bottom AUC score** | **-4** | **-3** | **-2** | **-1** | **0** | **1** | **2** | **3** |
| --- | --- | --- | --- | --- | --- | --- | --- | --- |
| Bottom 1/3 | 1 | 10 | 25 | 50 | 56 | 40 | 2 | 1 |
| Not in bottom 1/3 | 3 | 39 | 94 | 69 | 58 | 18 | 0 | 0 |

| **Middle AUC score** | **-2** | **0** | **1** | **3** |
| --- | --- | --- | --- | --- |
| Middle 1/3 | 2 | 130 | 6 | 0 |
| Not in middle 1/3 | 6 | 302 | 18 | 2 |

**Precision / Recall of the top AUC score**

| **Threshold** | **-5.5** | **-3** | **-0.5** | **0.5** | **1.5** | **2.5** | **3.5** |
| --- | --- | --- | --- | --- | --- | --- | --- |
| Precision (%) | 30.7 | 30.6 | 32.0 | 39.0 | 49.7 | 61.3 | 75.0 |
| Recall (%) | 100.0 | 99.3 | 96.5 | 83.2 | 69.2 | 34.3 | 4.2 |

**Precision / Recall of the bottom AUC score**

| **Threshold** | **-3.5** | **-2.5** | **-1.5** | **-0.5** | **0.5** | **1.5** | **2.5** |
| --- | --- | --- | --- | --- | --- | --- | --- |
| Precision (%) | 39.8 | 42.1 | 50.7 | 53.5 | 70.5 | 100.0 | 100.0 |
| Recall (%) | 99.5 | 94.1 | 80.5 | 56.6 | 23.2 | 1.6 | 0.5 |
